# ACIVY: An Enhanced IVY Optimization Algorithm with Adaptive Cross Strategies for Complex Engineering Design and UAV Navigation

**DOI:** 10.3390/biomimetics10070471

**Published:** 2025-07-17

**Authors:** Heming Jia, Mahmoud Abdel-salam, Gang Hu

**Affiliations:** 1 School of Information Engineering, Sanming University, Sanming 365004, China; 2 Faculty of Computers and Information Science, Mansoura University, Mansoura 35516, Egypt; 3 Department of Applied Mathematics, Xi’an University of Technology, Xi’an 710054, China

**Keywords:** Ivy optimization algorithm, UAV path planning, adaptive mutation, complex engineering

## Abstract

The Adaptive Cross Ivy (ACIVY) algorithm is a novel bio-inspired metaheuristic that emulates ivy plant growth behaviors for complex optimization problems. While the original Ivy Optimization Algorithm (IVYA) demonstrates a competitive performance, it suffers from limited inter-individual information exchange, inadequate directional guidance for local optima escape, and abrupt exploration–exploitation transitions. To address these limitations, ACIVY integrates three strategic enhancements: the crisscross strategy, enabling horizontal and vertical crossover operations for improved population diversity; the LightTrack strategy, incorporating positional memory and repulsion mechanisms for effective local optima escape; and the Top-Guided Adaptive Mutation strategy, implementing ranking-based mutation with dynamic selection pools for smooth exploration–exploitation balance. Comprehensive evaluations on the CEC2017 and CEC2022 benchmark suites demonstrate ACIVY’s superior performance against state-of-the-art algorithms across unimodal, multimodal, hybrid, and composite functions. ACIVY achieved outstanding average rankings of 1.25 (CEC2022) and 1.41 (CEC2017 50D), with statistical significance confirmed through Wilcoxon tests. Practical applications in engineering design optimization and UAV path planning further validate ACIVY’s robust performance, consistently delivering optimal solutions across diverse real-world scenarios. The algorithm’s exceptional convergence precision, solution reliability, and computational efficiency establish it as a powerful tool for challenging optimization problems requiring both accuracy and consistency.

## 1. Introduction

Optimization problems are ubiquitous across diverse domains of human endeavor, ranging from engineering design to resource allocation and scientific discovery [1]. These problems typically involve identifying optimal values for decision variables within specified constraints to minimize or maximize objective functions. As computational demands have evolved, stochastic methodologies have emerged as powerful alternatives to traditional deterministic approaches [2]. Among these, metaheuristic algorithms (MAs) have gained significant prominence due to their computational simplicity, robust optimization capabilities, and applicability across various practical engineering domains [3]. Unlike conventional optimization techniques, MAs demonstrate remarkable flexibility, offering broader search capabilities without imposing stringent requirements on the mathematical properties of objective functions [4].

MAs are broadly categorized into nature-inspired and non-nature-inspired methodologies [5,6]. While non-nature-inspired algorithms have contributed valuable optimization tools across multiple disciplines, they often exhibit limitations in practical implementations. Consequently, nature-inspired algorithms have gained dominance in optimization applications due to their superior search capabilities, minimal parameter requirements, and reduced problem-specific constraints [7]. Recent years have witnessed extensive applications of these algorithms across diverse fields, including robotics systems [8], image processing applications [9], big data analytics [10], energy prediction [11,12], human activity recognition [13,14], economic dispatch problems [15], feature selection methodologies [16,17,18], cloud scheduling [19], intrusion detection systems [20,21], chip design optimization [22], engineering optimization problems [23,24], wireless sensor networks [25], and other complex operational environments [26,27].

Nature-inspired algorithms derive their foundations from different natural phenomena and are classified into swarm intelligence algorithms (SIAs), evolutionary algorithms (EAs), and physics-based algorithms (PBAs). SIAs emulate collective behaviors observed in natural systems and represent the most diverse category of nature-inspired methodologies. Some representative SIAs include grey wolf optimization (GWO) [28], particle swarm optimization (PSO) [29], Harris Hawks Optimization (HHO) [30], whale optimization algorithm (WOA) [31], Marine Predators Algorithm (MPA) [32], sparrow search algorithm (SSA) [33], Northern Goshawk Optimization (NGO) [34], termite life cycle optimizer (TLCO) [35], and others. EAs are founded on Darwinian evolutionary principles and encapsulate biological evolution mechanisms. Classical EAs encompass differential evolution (DE) [36], the genetic algorithm (GA) [37], the quantum-inspired evolutionary algorithm (QEA) [38], the covariance matrix adaptive evolutionary strategy (CMA-ES) [39], and other related variants. PBAs are optimization opus cases, modeling the underlying physical or mathematical laws discovered by scientists. Examples of PBAs are the sine cosine algorithm (SCA) [40], gravitational search algorithm (GSA) [41], K-means optimizer (KO) [42], and atom search optimization (ASO) [43].

Despite demonstrating a strong competitive performance in addressing complex practical optimization challenges, nature-inspired algorithms remain susceptible to premature convergence toward local optima due to inherent performance limitations. The no free lunch (NFL) theorem establishes that no single algorithm can consistently outperform all other intelligent algorithms across every application domain [44]. Nevertheless, to develop efficient and universally applicable optimization methodologies for practical applications, researchers continuously enhance original algorithms to achieve more reliable solutions across diverse problem categories. Common enhancement approaches include hybridization with complementary algorithms and the incorporation of strategies based on different optimization principles. For instance, Meng et al. developed improved DE variants by integrating distinct mutation strategies for global numerical optimization [45]. Gao and Zhao introduced VW-GWO, a GWO variant incorporating variable weights based on the correlation between grey wolf positions and their hierarchical status [46]. Das et al. developed IPSO-IGSA through the fusion of enhanced PSO with improved GSA, applying it to multi-robot optimal path determination in cluttered environments [47]. Naik et al. addressed multi-layer threshold segmentation based on normalized squared variance using their proposed leader slime mould algorithm (LSMA) [48]. To address the slow convergence limitations of the firefly algorithm (FA), Ball et al. developed an improved FA (IFA) for optimizing drop ejection frequency in EHD inkjet printing systems [49]. Gharehchopogh and Abdollahzadeh introduced an improved HHO (IHHO) incorporating random-key encoding for traveling salesman problem solutions [50]. Akopov introduced a novel Matrix-Based Hybrid Genetic Algorithm (MBHGA) that integrates real-coded and matrix binary-coded crossover operators to optimize symmetric strategies in an agent-based model simulating firm behaviors under trade restrictions [51]. Madushani and Kasthurirathna extended the genetic algorithm that integrates Evolutionary Game Theory-based strategy adoption into GA-driven multi-agent systems. The key contribution lies in enhancing GA with socially inspired learning dynamics, enabling more adaptive and cooperative agent behavior for applications like multi-robot navigation [52]. Wang et al. introduced the Crisscross Harris Hawks Optimizer (CCHHO), a novel variant of HHO enhanced with crisscross optimization strategies to improve global exploration and avoid local optima. By integrating vertical and horizontal crossover along with a competitive operator, CCHHO effectively balances exploration and exploitation [53]. Chakraborty proposed the HCCWOA algorithm, an enhanced version of the whale optimization algorithm that integrates horizontal crossover, cooperative learning, and inertia weight to improve both exploration and exploitation capabilities. Designed for feature selection, HCCWOA operates in a wrapper framework with KNN, offering a more effective mechanism [54]. Additionally, Izci and Ekinci enhanced original algorithms through hybridization with complementary algorithms and improved strategy integration, applying these enhanced intelligent algorithms to vehicle cruise control system optimization [55,56].

Drawing inspiration from the climbing and spreading behaviors of ivy plants, this paper introduces a novel bio-inspired MA. Natural ivy demonstrates remarkable adaptive growth patterns, initially spreading horizontally across surfaces to locate suitable support structures, then transitioning to vertical climbing toward optimal light sources, and subsequently expanding around favorable positions to maximize resource acquisition [57]. This sophisticated biological strategy provides an excellent metaphor for optimization processes, where solutions must balance exploration of new regions, exploitation of promising areas, and local refinement around optimal zones. The Ivy Optimization Algorithm (IVYA) emulates these natural behaviors through coordinated growth phases, neighbor-guided climbing mechanisms, and adaptive spreading strategies around elite solutions. However, preliminary investigations revealed that IVYA, while demonstrating competitive performance, exhibits limitations in inter-individual information exchange, directional guidance for escaping local optima, and smooth transitions between exploration and exploitation phases. To address these shortcomings, this work presents the Adaptive Cross Ivy (ACIVY) algorithm, which incorporates three strategic enhancements, including the crisscross strategy for improved population interaction, the LightTrack strategy for enhanced directional guidance, and the Top-Guided Adaptive Mutation for refined exploration–exploitation balance.

To validate the optimization capabilities of ACIVY, comprehensive evaluations are conducted against established algorithms across multiple benchmark suites and practical engineering applications. Experimental results demonstrate that ACIVY achieves superior performance compared to competing algorithms across the majority of test functions, consistently identifying optimal design solutions across diverse optimization scenarios. In summary, the main contributions of this paper are as follows:

- To overcome the shortcomings of the original IVYA algorithm, a novel optimization algorithm named ACIVY is proposed by introducing the crisscross strategy, the LightTrack strategy, and the Top-Guided Adaptive Mutation strategy.
- Classical, recently proposed, and improved intelligent algorithms are selected as comparison algorithms. The optimization capability of ACIVY is evaluated on the CEC2017 test set in 50 dimensions and the CEC2022 test set in the highest dimension. The computational results of various performance indicators demonstrate that the proposed ACIVY achieves the best overall performance across most test functions.
- ACIVY is applied to engineering design optimization problems, structural design challenges, and UAV path planning applications. The results show that ACIVY can consistently provide the most reliable optimal design strategies for diverse practical problems.

The remainder of this paper is structured as follows: Section 2 presents the fundamental principles and mathematical formulation of the original Ivy Optimization Algorithm along with the problem statement. Section 3 introduces the enhanced ACIVY algorithm through a detailed description of the crisscross strategy, LightTrack strategy, and Top-Guided Adaptive Mutation mechanisms. Section 4 establishes the various applications of ACIVY including experimental methodology on CEC2017 and CEC2022 benchmark, practical applicability through engineering design optimization and ACIVY for UAV path planning applications. Finally, Section 5 provides conclusions and discusses future research directions.

## 2. Ivy Optimization Algorithm (IVYA) and Problem Statement

IVYA is inspired by the natural growth cycle of ivy plants, especially their adaptive climbing and spreading mechanisms in response to environmental constraints. Ivy, as a creeping plant, begins its growth horizontally across the ground surface in search of support. Once a structure such as a rock, tree, or wall is encountered, the plant anchors itself and begins vertical growth, climbing towards sunlight. This dynamic behavior illustrates a balance between two fundamental strategies: exploration (seeking viable attachment structures) and exploitation (climbing toward better growth conditions).

### 2.1. Phase 1: Population Initialization

The objective of this phase is to initialize a diverse set of candidate solutions across the entire decision space. The initial positions of the solutions $X_{i}$ are generated using the following equation:

(1) $$X_{i}=LB+rand\left(1,D\right)⊙\left(UB-LB\right),i=1,2,\ldots ,N_{pop}$$

In this equation, $X_{i}$ represents the position vector of the i-th ivy plant (solution) in a D-dimensional decision space. The vector rand (1,D) consists of D independent random numbers uniformly distributed in the interval [0,1]. LB and UB are the vectors representing the lower and upper bounds of each decision variable, respectively. The operator ⊙ denotes elementwise multiplication, allowing the random scaling of search ranges for each variable independently. $N_{\mathrm{pop}}$ defines the total number of solutions in the population.

### 2.2. Phase 2: Coordinated Growth of Ivy Solutions

The goal of this phase would be to model exploratory behavior in which solutions self-regulate their velocity of growth through time to prevent stagnation and maintain adaptability. The update of growth velocity is defined as follows:

(2) $$\Delta Gv_{i}(t+1)={rand}^{2}⊙\left(N(1,D)⊙\Delta Gv_{i}(t)\right)$$

In this case, $\Delta Gv_{i}(t)$ is the present growth velocity of the i-th solution at iteration t and $\Delta Gv_{i}(t+1)$ is the new velocity at iteration t+1. The vector N(1,D) is made up of D random numbers having a standard normal distribution with a mean of zero and standard deviation of one. The term ${rand}^{2}$ is a scalar random variable distributed according to the probability density function $f(x)=\frac{1}{2\sqrt{x}}$, which favors smaller values, thus encouraging finer exploration around the current position. The operator ⊙ ensures that the update is applied elementwise across all dimensions.

### 2.3. Phase 3: Climbing Toward Light

This phase models ivy’s directional climbing when it discovers a viable surface or support. In IVYA, each solution identifies a nearby fitter solution and uses it as a reference point to guide its climb.

Let $X^{S}=\left[X_{1}^{S},X_{2}^{S},\ldots ,X_{N_{\mathrm{pop}\;}}^{S}\right]$ be the population sorted in ascending order of fitness. The best solution is denoted as $X_{\mathrm{Best}\;}=X_{1}^{S}$. For a given solution $X_{i}$, the guiding neighbor $X_{ii}$ is chosen as

(3) $$X_{ii}=\left\{\begin{array}{ll} X_{j-1}^{S}, & \;\mathrm{if}\;X_{i}=X_{j}^{S}\;\mathrm{and}\;X_{i}\neq X_{\mathrm{Best}\;}\\ X_{i}, & \;\mathrm{if}\;X_{i}=X_{\mathrm{Best}\;} \end{array}\right.$$

Once $X_{ii}$ is identified, the new position is calculated using

(4) $$X_{i}^{\mathrm{new}\;}=X_{i}+\left|N\left(1,D\right)\right|⊙\left(X_{ii}-X_{i}\right)+N\left(1,D\right)⊙\Delta Gv_{i}$$

Here, |N(1,D)| is the absolute value of a normally distributed vector, ensuring that the climbing direction maintains a forward bias. The term ($X_{ii}-X_{i}$) computes the vector displacement from the current solution to the stronger neighbor. Then, the growth velocity is also updated dynamically as follows:

(5) $$\Delta Gv_{i}=\left\{\begin{array}{ll} X_{i}⊘(UB-LB), & \;\mathrm{if}\;t=1\\ {rand}^{2}⊙\left(N(1,D)⊙\Delta Gv_{i}\right), & \;\mathrm{if}\;t\;>1 \end{array}\right.$$

In this equation, ⊘ denotes elementwise division.

### 2.4. Phase 4: Spreading Around the Best Solution

After the ivy has established a good foothold, it will then start to grow outward to cover greater territory and to increase its chances of survival and reproduction. In IVYA, this stage corresponds to increased local search of the best-known solution. All the solutions are drawn towards the globally best solution, but with randomized perturbations to search its local vicinity. The new position of each solution is calculated as

(6) $$X_{i}^{\mathrm{new}\;}=X_{\mathrm{Best}\;}⊙\left(rand(1,D)+N(1,D)⊙\Delta Gv_{i}\right)$$

In this formulation, $X_{\mathrm{Best}\;}$ is the current best solution. The vector rand(1,D) introduces stochastic diversity around the best solution. The growth velocity is recalculated using

(7) $$\Delta Gv_{i}^{\mathrm{new}\;}=X_{i}^{\mathrm{new}\;}⊘(UB-LB)$$

### 2.5. Phase 5: Adaptive Survivor Selection

This stage controls the evolutionary choice mechanism that ivy uses to alternate growth strategies. The algorithm determines whether a solution should keep searching (climb) or exploit (spread) depending on its comparative performance with respect to the best-known solution. An adaptive threshold β is defined as follows:

(8) $$\beta =\frac{2+rand}{2}$$

If the fitness $f\left(X_{i}\right)$ of a solution is less than $\beta \cdot f\left(X_{\mathrm{Best}\;}\right)$, the solution is deemed promising and undergoes the spreading phase (Phase 4). Otherwise, it re-enters the climbing phase (Phase 3) for further refinement. After generating the new population $X^{\mathrm{new}\;}$, it is merged with the existing population:

(9) $$X^{\mathrm{Merged}\;}=concat(X,X^{new})$$

This results in a merged pool of $2N_{\mathrm{pop}\;}$ solutions. These are sorted by fitness to produce

(10) $$X^{\left(M/S\right)}=\left[X_{1}^{\left(M/S\right)},X_{2}^{\left(M/S\right)},\ldots ,X_{2N_{\mathrm{pop}\;}}^{\left(M/S\right)}\right]$$

Finally, the top $N_{\mathrm{pop}\;}$ individuals are selected to proceed to the next iteration:

(11) $$X=\left[X_{1}^{\left(M/S\right)},X_{2}^{\left(M/S\right)},\ldots ,X_{N_{pop}}^{\left(M/S\right)}\right]$$

### 2.6. Problem Statement

The initial IVYA algorithm, as biologically inspired as it was and as effective in balancing between climbing and spreading behaviors, has several limitations that impede its behavior on complex optimization tasks. Most prominent among them are the fact that it does not have a structured inter-individual exchange of information, does not have long-range guidance away mechanisms to get out of local optima, and lacks a smooth exploitation–exploration transition. Furthermore, the original IVYA suffers from inadequate population diversity maintenance during the optimization process, where solutions tend to cluster around locally optimal regions without sufficient mechanisms to preserve genetic variation across the search space, leading to premature convergence and reduced exploration capability in multimodal optimization landscapes. Additionally, the algorithm’s directional search strategy lacks adaptive memory components that could leverage historical search information to guide future exploration, resulting in repetitive search patterns and inefficient navigation through complex multi-dimensional fitness landscapes, particularly in high-dimensional optimization scenarios where traditional climbing and spreading behaviors become insufficient for comprehensive space coverage and optimal solution discovery.

## 3. Adaptive Cross Ivy (ACIVY)

This section proposes three main strategies used to solve the limitations of the original IVYA. The four main strategies include the crisscross strategy (CCS), LightTrack (LT) strategy, and Top-Guided Adaptive Mutation (TGAM) strategy.

### 3.1. Crisscross Strategy (CCS)

One of the core challenges encountered in the original IVYA lies in its limited capability to effectively exchange information among the population members and insufficient dimensionwise adaptation within individual solutions. While IVYA’s climbing and spreading phases promote guided movement, they often lack the structural diversity needed to jump across stagnated regions of the search space, particularly in high-dimensional landscapes. This increases the risk of the algorithm converging prematurely to suboptimal zones due to insufficient exploration among individuals and within their internal components.

To address this, a crisscross-based enhancement is integrated into ACIVY. This mechanism mimics a two-fold search dynamic: it introduces lateral (horizontal) information sharing among individuals and internal (vertical) recombination across dimensions of the same individual [58]. These two operations are known as Horizontal Crossover Search (HCS) and Vertical Crossover Search (VCS), respectively.

In HCS, two distinct individuals, say $X_{i}$ and $X_{k}$, are chosen in the population and a crossover is carried out on a particular dimension j to give new candidate positions. The mathematical formulas to direct this operation are

(12) $$H_{i}^{j}=r_{1}\cdot X_{i}^{j}+\left(1-r_{1}\right)\cdot X_{k}^{j}+c_{1}\cdot \left(X_{i}^{j}-X_{k}^{j}\right)$$

(13) $$H_{k}^{j}=r_{2}\cdot X_{k}^{j}+\left(1-r_{2}\right)\cdot X_{i}^{j}+c_{2}\cdot \left(X_{k}^{j}-X_{i}^{j}\right)$$

Here, $X_{i}^{j}$ and $X_{k}^{j}$ represent the values in the j-th dimension of individuals i and k. The weights $r_{1}$ and $r_{2}$ are random scalars independently and uniformly selected in [0,1], which adds stochasticity to the weighted sums of the parents. Meanwhile, $c_{1}$ and $c_{2}$ are drawn uniformly in the interval [−1,1], which enables both contraction and expansion in differential recombination. The values $H_{i}^{j}$ and $H_{k}^{j}$ are the offspring as a result of this crossover. After creation, a greedy selection process is applied to see which parents and offspring are the fittest, and only the best candidates are kept, maintaining the quality of the population.

In VCS, a single individual $X_{i}$ undergoes recombination within itself. Two dimensions j1 and j2 are chosen at random from the solution vector. These two components are blended to generate a new dimension $V_{i}^{j}$ as follows:

(14) $$V_{i}^{j}=r_{3}\cdot X_{i}^{j1}+\left(1-r_{3}\right)\cdot X_{i}^{j2}$$

Here, $r_{3}$ is a random value drawn from [0,1], ensuring that the offspring dimension is a convex combination of the two selected ones. $X_{i}^{j1}$ and $X_{i}^{j2}$ are the values of the selected dimensions. The resulting value $V_{i}^{j}$ replaces one of the parent dimensions, and similar to HCS, the update is accepted if it improves the individual’s overall fitness.

The HCS strategy improved the global search ability through horizontal crossover, which provides long-range exploration among agents. On the other hand, VCS enhances local search by encouraging fine-grained tuning in the structure of an individual. Combined, they constitute a complementary strategy that unifies the inter-agent cooperation and intra-agent refinement. The integration of the crisscross strategy significantly improves ACIVY’s ability to traverse rugged and multimodal landscapes.

### 3.2. LightTrack (LT) Strategy

In the original IVYA algorithm, although local exploitation and neighbor-driven climbing help solutions to progress, individuals may become confined within deceptive valleys of the search space. Once a moderately good area is encountered, the spreading and climbing mechanisms may no longer provide sufficient momentum to guide individuals out of misleading or low-fitness regions. This stagnation reduces search efficiency, particularly in complex multimodal functions.

The LT strategy draws inspiration from the orientation behavior of dung beetles, which rely on sunlight to maintain a straight path while rolling dung balls [59]. This enhancement helps the algorithm maintain guided movement while escaping unfavorable zones. The new position for the i-th solution is computed using the following equation:

(15) $$X_{i}^{new}(t+1)=X_{i}(t)+\alpha \cdot k\cdot X_{i}(t-1)+b\cdot \Delta x$$

In this expression, $X_{i}(t)$ represents the current position of the i-th candidate at the present iteration t. The term $X_{i}(t-1)$ corresponds to its previous position at iteration t−1, introducing a directional memory component. The scalar α takes a value of either +1 or −1, where +1 maintains the current trajectory and −1 introduces a deliberate reversal to simulate environmental disturbance. The coefficient k lies within the open interval (0,0.2], acting as a directional attenuation factor that controls the magnitude of the influence from the prior direction. The constant b is a positive number within (0,1) and regulates the degree of repulsion from poor-performing zones. Finally, the term Δx is defined as

(16) $$\Delta x=\left|X_{i}(t)-X_{\mathrm{Worst}\;}\right|$$

Here, $X_{Worst}$ represents the worst individual in the population based on objective function value.

Following the position update, the algorithm evaluates which candidate position should be retained. It compares the newly generated solution $X_{i}^{new}$ against another position $X_{i}(t+1)$ (which could be derived from IVYA’s standard mechanisms). The position with better fitness is retained using the following rule:

(17) $$X_{i}(t+1)=\left\{\begin{array}{ll} X_{i}^{\mathrm{new}\;}(t+1), & \;\mathrm{if}\;f\left(X_{i}^{\mathrm{new}\;}\right)<f\left(X_{i}\right)\\ X_{i}(t), & \;\mathrm{otherwise}\; \end{array}\right.$$

This decision-making process guarantees that directional guidance via LT is only accepted when it provides actual performance benefits. Integrating the LT strategy into ACIVY provides a robust mechanism for long-distance movement without blindly randomizing trajectories. By combining historical direction ($X_{i}(t-1)$) and a repulsive component from poorly performing areas ($X_{Worst}$), the strategy increases the algorithm’s capability to avoid fitness traps.

### 3.3. Top-Guided Adaptive Mutation (TGAM) Strategy

In the original IVYA algorithm, despite its use of local climbing and spreading behaviors, the transition from broad search (exploration) to focused refinement (exploitation) lacks a mechanism that gradually shifts attention toward higher-quality regions. Specifically, newly generated solutions are formed without any selective preference or directional bias toward better-performing areas, which can reduce convergence speed and precision in later stages of the optimization process.

To address this limitation, the TGAM strategy introduces an adaptive mutation scheme inspired by ranking-based guidance. Instead of relying solely on the current or global best solutions, each solution selectively mutates toward one of the top-performing candidates in the population. This selection pool, referred to as the “top S solutions” is dynamically adjusted as the algorithm progresses, beginning with a broad set of promising candidates and narrowing over time. The new candidate solution $X_{i}^{new}$ is generated using the following equation:

(18) $$X_{i}^{new}=X_{sbest}+F\cdot \left(X_{r1}-X_{r2}\right)$$

In this expression, $X_{sbest}$ represents a randomly chosen solution from the top S X N individuals in the population, where N is the total population size. This value differs from a deterministic best and introduces variation in the search direction. The variables $X_{r1}$ and $X_{r2}$ denote two different individuals selected at random from the entire population such that r1≠r2 and both are different from the current agent. To ensure that exploration is emphasized early and exploitation takes precedence in later iterations, the selection size S is updated dynamically using the following equation:

(19) $$S_{t}=1-\left(1-\frac{1}{N}\right)\cdot \frac{t-1}{T-1}$$

Here, $S_{t}$ represents the adaptive control parameter at iteration t. The variable T is the total number of iterations, and N is the number of solutions in the population. The above formulation guarantees that $S_{t}$ begins near 1 so that any solution amid the high-ranked candidates can be chosen to undergo mutation. Smoothly decreasing with the advancement of the algorithm, $S_{t}$ shrinks the selection pool diversity, focusing the mutation effort around the individuals with high fitness.

**Figure 1:**
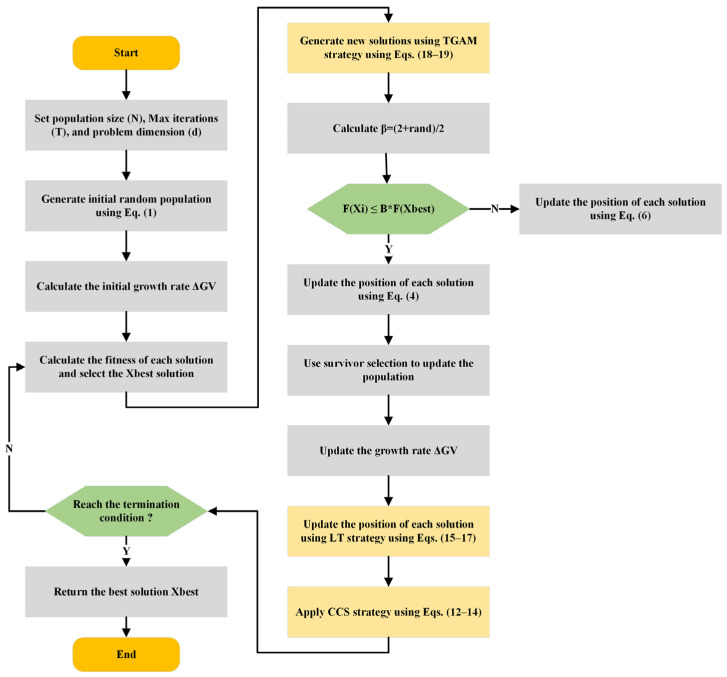
The proposed ACIVY.

The design provides two different working stages. The mutation mechanism promotes broad search early in the run, with a larger S, and thus a higher probability of drawing on a broad range of top contenders. Conversely, in later periods a lesser S causes the mutation to be skewed to the most elite areas, favoring more intensive exploitation. The incorporation of the TGAM strategy significantly strengthens the dynamic search control of ACIVY. During initial iterations, the increased diversity generated from a broad set of top-ranked solutions allows the population to probe a wider region of the search space, enhancing global exploration. As S contracts with time, the algorithm naturally focuses its effort around the most promising areas, improving its convergence accuracy. Thus, the main steps of ACIVY are shown in Figure 1 and Algorithm 1.
**Algorithm 1:** ACIVY algorithm**Input:** Maximum number of iterations T, Population size N, upper ub, lower bound lb.
**Output:** Optimal solution $X_{best}$, fitness value of optimal solution $f_{best}$.1.Initialize the initial population $X_{i}$, i←1,2,….,N using Equation (1)2.Compute the fitness value $f(X_{i})$ of each solution3.Save the best solution as the optimal one $X_{best}$
4.while t≤T do5.    for i=1 to N do6.        Generate new solution $X_{new}$ using TGAM strategy using Equations (18) and (19)7.        if $f\left(X_{new}\right)<f(X_{i})$ then8.            
$f\left(X_{i}\right)=f(X_{new})$
9.        end if10.    end for11.    for i=1 to N do12.        Calculate $\beta =1.5-0.5(\frac{t}{T})$
13.            if $f\left(X_{i}\right)\leq \beta \times f(X_{best})$ then14.                Update $X_{i,\;new}\;$ solution using Equation (4)15.            Else16.                Update $X_{i,\;new}$ solution using Equation (6)17.            end if18.            Calculate the fitness of the newly generated solution $X_{i,\;new}$.19.            Update $\Delta Gv_{i}$ using Equation (5)20.            Add the new solution $X_{i,new}$ as i-th component $X_{new}$21.    end for22.    Merge the populations X and $X_{new}$23.    Calculate the fitness values of the new combined population and select the top N solutions24.    for i=1 to N do25.        Update the position of each solution using LT strategy using Equations (15)–(17)26.    end for27.    Apply CCS strategy using Equations (12)–(14)28.    
t=t+1
29.end while30.Return $X_{best}$ and its fitness value $f_{best}$

### 3.4. Computational Complexity Analysis

The computational complexity of an optimization algorithm reflects how its processing time and memory usage scale with key problem variables. For both the original IVYA and the enhanced ACIVY algorithms, complexity is determined by three primary parameters: the population size N, the number of decision variables or dimensions D, and the maximum number of iterations T.

In the original IVYA, each solution in the population performs a position update based on either the climbing or spreading phase. These updates involve arithmetic operations across D dimensions. In each iteration, fitness values are evaluated for all N individuals, and the population is sorted based on fitness to maintain the best solutions. The position updates for the full population require O(N⋅D) operations per iteration. Evaluating the fitness function for each solution adds O(N), and sorting the population introduces O(Nlog N). However, in high-dimensional settings where D is large, the dominant cost is O(N⋅D), making the total time complexity across all iterations O(T⋅N⋅D). The memory usage is mainly attributed to storing positions, velocities, and fitness scores for all individuals, resulting in a space complexity of O(N⋅D).

The enhanced version, ACIVY, incorporates three additional mechanisms including CCS, LT, and TGAM to improve the balance between exploration and exploitation. These strategies introduce additional operations per iteration, but they all remain linear in the population size and dimensionality. The CCS requires arithmetic operations over selected dimensions for each solution, which scales as O(N⋅D) per iteration. The LT strategy introduces a directional update contributing a cost O(N⋅D). The TGAM strategy introduces differential mutations resulting in O(N⋅D) cost per iteration.

Overall, the time complexity of ACIVY remains in the order of O(T⋅N⋅D), matching that of the original IVYA. The space complexity is also O(N⋅D), as the algorithm still stores the same set of solution vectors and associated information, with minor temporary vectors used during crossover or mutation operations that do not affect the overall memory footprint.

Finally, despite ACIVY proposing several strategic improvements to enhance the performance, it does not raise the order of computational complexity. Compared to the original IVYA, the algorithm has identical time and space scalability characteristics, but much faster convergence and more accurate solutions on a variety of optimization problems.

## 4. Application of the ACIVY Algorithm

### 4.1. ACIVY for Global Optimization

This section gives a thorough analysis of the ACIVY algorithm by describing the experimental setup, as well as examining its performance in comparison to the other optimization techniques. The analysis consists of extensive experimentation on the CEC2017 [60] and CEC2022 [61] benchmark functions, modeling various real-world optimization problems. The convergence dynamics of ACIVY, as well as the distribution of performance depicted by boxplots, are also discussed in this part. Further, comparative experiments against state-of-the-art evolutionary strategies are also provided to demonstrate the efficacy of ACIVY in handling high-dimensional and complicated optimization landscapes.

#### 4.1.1. Experimental Configuration and Settings

Two well-known benchmark sets, CEC2017 and CEC2022, were used to test ACIVY’s optimization power. CEC2017 suite comprises 29 different functions of different characteristics- unimodal (F1 and F3), multimodal (F4–F10), hybrid (F11–F20), and composite types (F21–F30). In the meantime, CEC2022 concentrates on 12 advanced functions designed to test the performance of algorithms on deceptive as well as high-dimensional problems. Each function of both suites is defined in the domain [−100,100]. These testbeds are designed purposefully to test the skill of an optimizer on both exploration (global search) and exploitation (local refinement).

Moreover, the experimental run was conducted with the dimensions of 50 for CEC2017 and 20 for CEC2022. All the tests were performed with a constant population size of 30 solutions, 30,000 function evaluations, and 30 repetitions were carried out independently to make the statistical analysis meaningful. All performances were conducted on a computing system composed of a 2.60 GHz Intel Core i7 10750H CPU, 32 GB RAM, and Windows 10 (64-bit), and all codes were written in MATLAB R2020b.

For benchmarking purposes, ACIVY was assessed against a wide spectrum of MAs, categorized into three main groups. The first group includes foundational methods such as AOA [62], WOA [31], and HHO [30], all widely adopted for their general reliability. The second group contains more recently introduced optimizers like SWO [63], COA [64], CFish [65], SHO [66], GJO [67], and HMO [68], which propose recent strategies in search methodology. The third group comprises advanced and competition-winning algorithms such as eCOA [69], CMAES [39], GWO_CS [70], RDWOA [71], CSOAOA [72], DAOA [73], ISSA [74], jDE [75], and IPSO_IGSA [76], representing state-of-the-art optimization capability. Detailed settings for each algorithm are listed in Table 1.

The selected benchmark algorithms to compare with are based on their proven relevance and confirmed efficiency in a wide range of optimization cases. The classical methods act as a solid background, the results of which are predictable over time, and the newer models represent the rising opinions and search strategies [77,78]. Additionally, newer and high-scoring algorithms, particularly those that have succeeded in competition, are included to ensure that the comparison framework spans a large performance range. This multi-level choice offers the opportunity to evaluate the performance of ACIVY carefully and objectively against popular, new, and state-of-the-art metaheuristics.

#### 4.1.2. Metrics for Evaluating Optimization Performance

In order to impartially assess the quality of work of the ACIVY algorithm among these different rivals, there are a number of statistical and descriptive measures. These are the average quality of solutions (AVG), standard deviation (SD), Friedman ranking (FR), and the Wilcoxon rank-sum significant test. All the metrics have their role in the profiling of consistency, reliability, and superiority of the optimizer.

- **Mean Fitness (AVG):** The measure of the quality of solutions typically attained is the average fitness score over different independent runs. This measurement is useful in the evaluation of the correctness and general performance of an algorithm in repetitive usage, within the same setup. It is calculated with

(20) $$AVG=\frac{1}{N}\sum_{i=1}^{N}\;f_{i}$$

where N is the number of runs and $f_{i}$ is the fitness value obtained in the $i^{th}$ trial.

- **Standard Deviation (SD):** SD quantifies the extent of dispersion of the fitness values around the mean, providing information about the consistency and stability of the results produced by the algorithm. Smaller variations show that the optimizer provides consistent results when repeated many times. It can be calculated as follows:

(21) $$SD=\sqrt{\frac{1}{N}\sum_{i=1}^{N}\;\;{\left(f_{i}-AVG\right)}^{2}}$$

- **Friedman Ranking (FR)** [79]: This non-parametric statistical test ranks algorithms based on their relative performances across multiple problem instances. A lower average rank suggests a superior performance. The final ranking is derived from averaging ranks over all tested functions. The Friedman test statistic is then evaluated using a chi-squared distribution to determine consistency in relative performance across functions.
- **Wilcoxon Rank-Sum Test** [80]: To establish whether performance differences between ACIVY and any competing algorithm are statistically meaningful, the Wilcoxon rank-sum test is utilized. A p-value below 0.05 denotes a significant difference. If ACIVY achieves better results, it is marked with R+; if no clear difference exists, it is annotated with R=; and if ACIVY underperforms, it is labeled with R−.

A combination of these metrics provides an extensive basis to compare ACIVY to other algorithms and make sure that all findings regarding the optimization performance of this algorithm are statistically aligned and indicative of its practical potential.

#### 4.1.3. Results Discussion Using CEC2022

The main goal of this part is to compare and review the functionality of the proposed ACIVY algorithm with an extensive list of well-known optimization algorithms on the CEC2022 benchmark collection. The variety of tested algorithms embodies the various algorithmic paradigms and serves as a thorough testbed to evaluate the performance of ACIVY on a wide range of optimization problems as presented in Table 2.

On the unimodal function F1, ACIVY shows an outstanding performance, having the best rank and averaged fitness of 300.367, with an extremely low standard deviation of 0.354. Such a remarkable performance implies that ACIVY converges more precisely and reliably in single-optimum landscapes. The algorithm has spectacularly beaten all competitors, with the second-best performer (HHO) having a fitness value. Such a steep rise in performance underlines how well ACIVY’s coordinated growth and adaptive mechanisms exploit unimodal search spaces. Over the multimodal functions, ACIVY shows a continuously high performance and takes the leading position on F2, F3, and F4. On F2, ACIVY attains an average fitness of 454.503, only slightly higher than the original IVY at 461.213, indicating the improvement made with ACIVY along with preserving the strengths of the core algorithm. In the case of F3, ACIVY attains the theoretical maximum of 600.000 with a standard deviation of zero, indicating absolute convergence and superb reliability. Such a huge improvement suggests that ACIVY is more capable of traversing multimodal landscapes effectively and reliably finding global optima.

Furthermore, the hybrid functions reveal ACIVY’s robust adaptability across different problem characteristics. ACIVY secures first place on F5 and F7, second place on F6, and third place on F8, maintaining an average rank of 2 across this category. Notably, on F5, ACIVY achieves a fitness value of 1402.843, substantially outperforming the second-best GJO (1891.702). The algorithm’s performance on F6 is particularly noteworthy, where it achieves the second-best result with a significantly lower standard deviation compared to many competitors, indicating superior solution stability. In the most challenging composite function category, ACIVY demonstrates consistent excellence by securing first place on F9, F10, F11, and F12. This comprehensive dominance across all composite functions is particularly significant, as these problems combine multiple optimization challenges and represent the most complex scenarios in the benchmark suite. On F9, ACIVY achieves a near-perfect performance with a fitness value of 2480.811 and an exceptionally low standard deviation of 0.054, indicating both optimal solution quality and remarkable consistency across independent runs.

The base IVY algorithm shows decent results and ends up at a rank of 4, which confirms the base idea but also shows how much better the results can be with the additions that ACIVY provides. CFish takes the second overall spot with a solid performance across many functions, and GJO completes the top three with steady mid-range rankings. Such advanced algorithms as HHO, SHO, and HMO have different performance levels, where HHO took sixth place and provided competitive results on particular functions, such as F1. Nevertheless, the performance of all these established algorithms is not consistent with the performance of ACIVY on the wide categories of functions. Moreover, the algorithms ranked last (SWO, AOA, and COA) perform especially poorly on multimodal and composite functions, and their results have a high standard deviation, which means that the algorithms do not produce reliable solutions. This comparison underlines the superiority of ACIVY in terms of robustness and flexibility on varied optimization landscapes.

The behavior of the performance of ACIVY is distinguished not only by high-quality average fitness values but also by the low standard deviations in most functions. This combination implies that ACIVY reliably converges to high-quality solutions on multiple independent runs, an important trait in the practical use of optimization. The capability of the algorithm to sustain low variance with optimal or near-optimal fitness values shows the efficiency of adaptive mechanisms and well-balanced exploration–exploitation schemes.

Having the average rank of 1.25 over all twelve functions and obtaining the last rank of 1, ACIVY confirms itself as the most efficient algorithm in this full comparative study. The large difference in performance between ACIVY and the second-placed CFish (average rank 4.00) highlights the extensive algorithmic progress that the crisscross strategy, LT strategy, and TGAM improvements bring. These findings unreservedly show that the bio-inspired improvements incorporated in ACIVY effectively overcome the weaknesses of the original IVY algorithm, without affecting its principal advantages. The outstanding performance of the algorithm in a wide variety of categories of functions confirms its applicability to real-world optimization problems, which need robust, reliable, and high-accuracy solutions.

**Figure 2:**
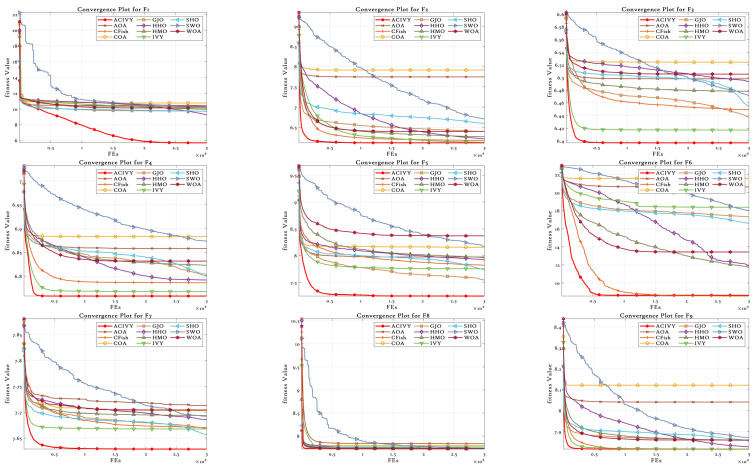

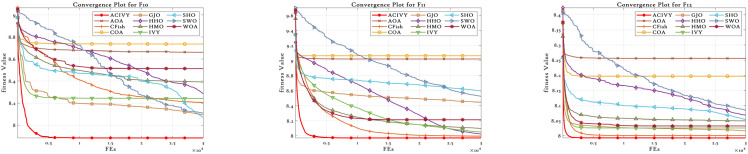
Convergence curves of different algorithms using CEC2022.

The convergence plots show the clear optimization behavior of ACIVY on the CEC2022 benchmark functions, and it exhibits better convergence properties than the competing algorithms as shown in Figure 2. According to Figure 2, ACIVY can be characterized by fast initial convergence and subsequent stable refinement stages and is fastest in terms of fitness values in all categories of functions. The convergence curve of the algorithm can be described as having very sharp initial drop-off curves followed by very rapid stabilization at optimal or near-optimal solutions, which implies the existence of effective exploration-to-exploitation transition mechanisms within the improved ivy-inspired strategies. In the case of unimodal and simple multimodal functions (F1–F4), ACIVY shows a remarkable convergence rate and achieves optimal solutions in the initial 5000–10,000 FEs. The convergence curves depict dramatic fitness gains during the initial iterations, where F1 and F3 converged to the theoretical optimum with little variance. Conversely, the competing algorithms either converge at non-optimal solutions or have slower rates of convergence with better final fitness values. It is noteworthy that the original IVY algorithm demonstrates decent convergence behaviors but cannot compete with ACIVY in terms of accuracy and speed, which proves the efficiency of the suggested improvements.

The hybrid functions (F5–F8) demonstrate the strong flexibility of ACIVY to a complex landscape, with identical convergence behavior when the problem complexity is increased. The algorithm shows no or minimal oscillations and no stagnation periods associated with state-of-the-art algorithms. Function F6 and F7 especially demonstrate the high performance of ACIVY, in which the algorithm quickly converges in less than 10,000 FEs, whereas other algorithms are still struggling with either slow convergence or plateau phenomena. These functions feel the impact of the LT strategy, as they allow proper escape out of local optima, which other algorithms get stuck in. In the most difficult composite functions (F9–F12), the convergence pattern of ACIVY is extremely regular and accurate, showing the fastest descent rates and being stable during the optimization process. F11 and F12 demonstrate the capability of the algorithm in finding optimal solutions in these complex multimodal landscapes, whereas ACIVY either gets stuck in local optima or exhibits unstable convergence behavior. The attribution of the TGAM strategy is especially reflected in these functions, which allow an easy transition between exploration and exploitation stages without affecting the quality of the convergence. Such a high convergence performance in all types of functions proves the efficiency of ACIVY as a strong and trustworthy optimization algorithm to handle complex real-world problems.

**Figure 3:**
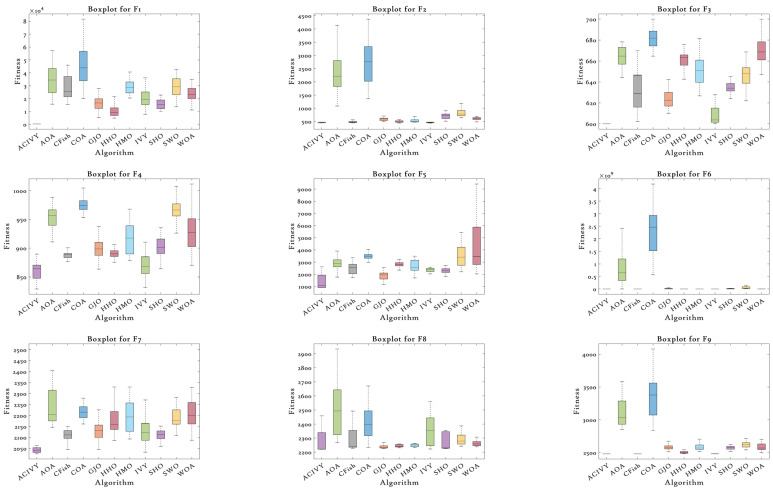

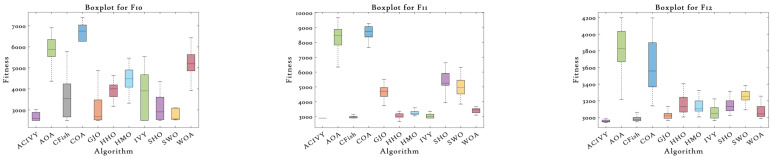
Boxplots of different algorithms using CEC2022.

On the other hand, the boxplot analysis shown in Figure 3 thoroughly reveals the statistical distribution nature and the reliability of the solution of ACIVY versus the rival algorithms on the CEC2022 benchmark functions. The boxplots display important performance metrics such as median fitness values, quartile ranges, the existence of outliers, and the overall consistency of solutions. ACIVY achieves statistical excellence in all aspects regarding medians, interquartile ranges, and the presence of outliers, showing the quality of the solution is optimal and the algorithm is very stable across all types of functions.

In the case of unimodal and multimodal functions (F1–F4), ACIVY shows the tightest boxplot distributions with the lowest median fitness values and minimal interquartile ranges. On F1, the boxplot of ACIVY is incredibly tight and centered around the theoretical optimum, whereas the competitors have much broader ranges and larger medians. The F3 boxplot especially shows the superiority of ACIVY, which has reached perfect convergence with zero variance, displayed as a single horizontal line at the optimal value of 600. Conversely, other algorithms, such as AOA and COA, exhibit very long whiskers and several outliers, which reflect the lack of consistency and reliability of the solution. The hybrid functions (F5–F8) illustrate the strong stability performance of ACIVY with regard to its boxplot structures that tend to be small even in multi-dimensional optimization landscapes. F6 shows that ACIVY has an outstanding performance with a very narrow distribution and low median, whereas competitors have large ranges and many outliers. The effect is especially dramatic when the compact boxplot of ACIVY is compared with the extremely wide distribution of COA spanning several orders of magnitude. F7 and F8 additionally affirm the statistical superiority of ACIVY, with low variance and ideal median rankings in comparison to the random and unreliable distributions of the rival algorithms. The composite functions (F9–F12) demonstrate the ultimate statistical excellence of ACIVY with the best median performance and superior distribution tightness. ACIVY ensures the minimum median values of all composite functions and shows an impressive consistency expressed in small boxplot ranges. This superiority is illustrated on F11, where ACIVY obtains a compact distribution with short whiskers, whereas the other algorithms, such as AOA and COA, exhibit a large population of outliers and broad quartile sticks. The statistical experiment appears to clearly indicate that ACIVY is not only capable of higher optimization performance but also offers the best degree of algorithm robustness and predictability which are critical properties of algorithms intended to be used in practice and needed to generate high-quality solutions consistently.

The testing of statistical significance is an important aspect in the optimization algorithm performance superiority proofs, as the observed performance gains can be easily attributed to random fluctuations [81]. The analysis of MAs must be accompanied by sound statistical testing in order to determine the confidence of comparative outcomes, especially in the case of stochastic optimization algorithms, which are by nature prone to variation with multiple independent runs. In this analysis, two basic non-parametric statistical tests are used: the Friedman test to evaluate the overall ranking and the Wilcoxon rank-sum test to compare each pair of algorithms, respectively, which were explicitly invented to work with non-normal distribution data, as optimization algorithm performance data often is.

**Figure 4:**
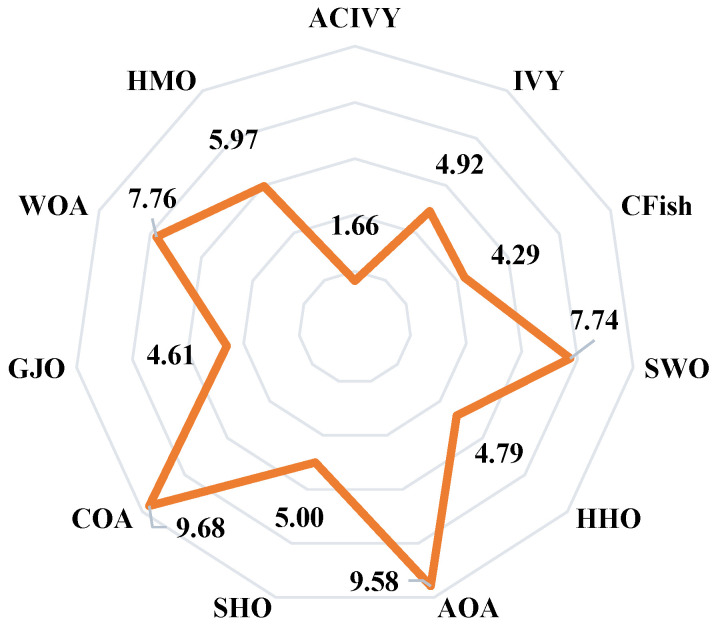
Friedman ranks various algorithms using CEC2022.

Figure 4 shows the results of the Friedman test, which gives a full ranking analysis of each of the algorithms on the entire CEC2022 benchmark suite. ACIVY attains the best average rank of 1.66 and significantly outcompetes all the competing algorithms and statistically dominates them in varying optimization landscapes. The original IVY algorithm still achieves a respectable average rank of 4.92, confirming the validity of the underlying approach but pointing to the immense gains made by ACIVY’s enhancements. The ranking order shows the existence of specific performance levels, where CFish (4.29) and GJO (4.61) comprise the second performance level and HHO (4.79) and SHO (5.00) belong to the middle-level performances. HMO (5.97), SWO (7.74), and WOA (7.76) are placed in the bottom part of the ranking, and AOA (9.58) and COA (9.68) take the last places. Such statistical ranking proves the outstanding ability of consistency in the performance of ACIVY in all categories of functions, and this shows its great flexibility in facing different kinds of optimization problems.

**Figure 5:**
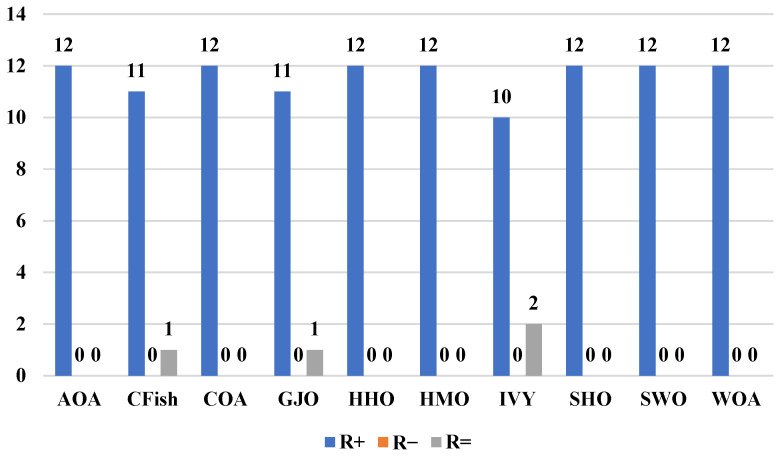
Wilcoxon test results for ACIVY versus other algorithms using CEC2022.

On the other hand, Figure 5 shows the pairwise Wilcoxon rank-sum test results, which extensively statistically prove the superiority of ACIVY over each of the competing algorithms in a pairwise manner. The analysis shows the clear statistical overpowering of ACIVY with higher performance (R+) values varying between 10 and 12 functions over all the competitors, whereas the R− values are always zero in all of the pairwise comparisons. Compared to AOA, CFish, COA, GJO, HHO, HMO, SWO, and WOA, ACIVY has statistical dominance (shows a better performance on 12 functions and never performs worse on any of the 12 functions). ACIVY also performs better on 10 functions against IVY and SHO, with 2 ties (R=), and once more, 0 worse performances. The R= values suggest that there are some statistical ties with CFish and GJO (one function each) and SHO (two functions), but these are not losses but statistical ties, which once again underline the superiority of ACIVY across the board.

This statistical evidence gives unquestioning confirmation of the algorithmic superiority of ACIVY over the benchmark suite. The Friedman test confirms the superior position of ACIVY with the minimal average rank of 1.66, and the Wilcoxon rank-sum test proves the overall superiority of the former with its pairwise wins. Overall, the lack of R− values (zero inferior performances) in all algorithmic comparisons is a startling statistical feat, which means that ACIVY does not show a worse performance than any of its competitors on any of the benchmark functions. This stable trend of statistical significance, together with the large performance gaps demonstrated by the Friedman ranks, gives strong indications that the improvements found by ACIVY are not fluctuations but actual algorithmic advances. The statistical strength of these findings puts high confidence in the reliability and efficacy of ACIVY, making it a statistically proven method of handling complex optimization problems where not only is excellent performance needed, but also reliability across a wide range of problem landscapes.

#### 4.1.4. Results Discussion with Advanced Algorithms Using CEC2017

In this section, the performance of ACIVY is compared with state-of-the-art algorithms on the CEC2017 benchmark suite of 50-dimensional problems, which, together with the earlier analysis on CEC2022, gives a thorough verification of the algorithm on various benchmark standards and problem dimensions. The comparative analysis involves ten advanced algorithms, namely, eCOA, CMAES, DAOA, CSOAOA, GWO_CS, RDWOA, jDE, ISSA, and IPSO_IGSA, which are recent advancements in evolutionary computation, swarm intelligence, and hybrid optimization techniques.

According to Table A1, ACIVY shows a remarkable performance advantage throughout the CEC2017 benchmark set, taking the final overall first rank with an incredible average rank of 1.41, which is significantly better than all other participating advanced algorithms. The algorithm ranks first on 20 of 29 functions (win rate of 69.0%), ranks second on 7 functions, third on F17, and fourth on F10, giving a top-two result on 93.1 percent of the benchmark problems. CSOAOA turns out to be the second-best-performing with the final rank of second (average rank 3.17), and GWO_CS ranks third (average rank 3.72), and eCOA ranks fourth (average rank 3.79). The performance hierarchy shows that there are large differences between ACIVY and its nearest rivals, where DAOA is placed at the bottom (final rank 10, average rank 10.00), indicating the existence of large algorithmic advantages of ACIVY. Function category analysis reveals ACIVY’s performance patterns across diverse optimization landscapes with distinct characteristics for each problem type. For unimodal functions (F1, F3), ACIVY achieves perfect dominance with a 100% win rate (two out of two functions), demonstrating exceptional exploitation capabilities where F1 shows a fitness value of 6296.553 compared to DAOA’s 2.83 × 10^11^. In multimodal functions (F4–F10), ACIVY secures first place on only two functions (F4 and F6), achieving a 28.6% win rate, but maintains a strong performance with second place on F5, F7, F8, and F9, and fourth place on F10. Despite the lower win rate, ACIVY’s fitness values remain highly competitive, with F4 showing 506.774 versus competitors ranging from 643.383 to 1.04 × 10^5^. The hybrid functions category (F11–F20) showcases ACIVY’s superior adaptability with an 80% win rate (8 out of 10), securing first place on F11, F12, F13, F14, F15, F16, and F18, with second-place finishes on F19 and third place on F17. In this category, F12 particularly exemplifies ACIVY’s dominance with a fitness value of 5.89 × 10^6^ versus DAOA’s 1.41 × 10^11^. For composite functions (F21–F30), ACIVY demonstrates remarkable consistency with 80% superiority (8 out of 10), winning on F20, F21, F23, F24, F25, F26, F27, F28, and F29, while achieving second place on F30 and F22. The performance distribution reveals ACIVY’s exceptional strength in complex hybrid and composite functions, where its enhanced mechanisms prove most effective.

The comparative analysis reveals significant fitness value disparities between ACIVY and advanced competitors across critical benchmark functions. On function F1, while ACIVY achieves 6296.553, its closest competitor, ISSA, reaches 1.76 × 10^8^, representing a large performance gap, with other algorithms like CMAES and DAOA performing orders of magnitude worse. Function F12 demonstrates even more dramatic differences, where ACIVY’s 5.89 × 10^6^ substantially outperforms eCOA’s 3.83 × 10^8^ and completely dominates DAOA’s catastrophic 1.41 × 10^11^. In the hybrid function F14, ACIVY maintains 1.78 × 10^5^ while advanced algorithms like CMAES struggle with 2.14 × 10^7^, and DAOA fails at 3.46 × 10^8^. These substantial performance margins highlight ACIVY’s superior convergence precision and global search capabilities compared to even the most sophisticated contemporary algorithms. Furthermore, ACIVY consistently achieves lower standard deviation values, indicating superior solution reliability compared to competitors that often exhibit high variance and inconsistent performance across independent runs.

**Figure 6:**
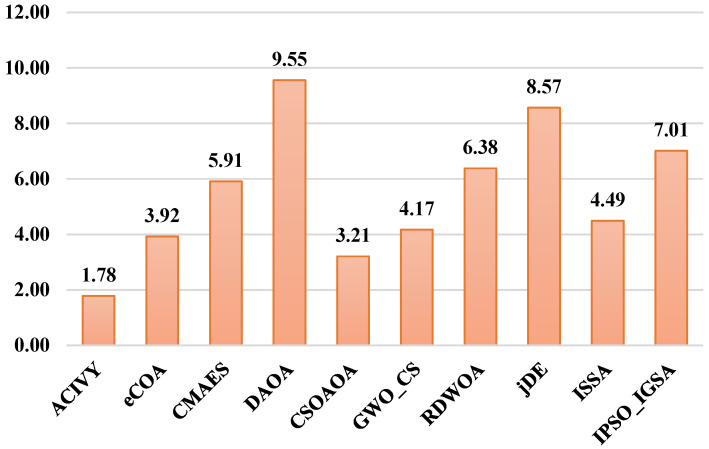
Friedman ranks different advanced algorithms using CEC2017, 50D.

The Friedman test statistical significance of the CEC2017 benchmark shown in Figure 6 generates the statistical dominance of ACIVY with a mean rank of 1.78, which is significantly better than all the sophisticated competitors on the 50-dimensional optimization search space. CSOAOA proves to be the most competitive algorithm with a mean rank of 3.21, slightly followed by eCOA 3.92, meaning that these algorithms are moderately competitive, irrespective of the fact that their performances differ significantly. There are the middle-tier GWO_CS (4.17) and ISSA (4.49), and the weak overall performance of such algorithms as CMAES (5.91), RDWOA (6.38), and IPSO_IGSA (7.01). The low-ranking algorithms, jDE (8.57) and DAOA (9.55), exhibit notably bad adaptation to high-dimensional optimization problems, where DAOA is ranked among the last in the majority of the functions.

**Figure 7:**
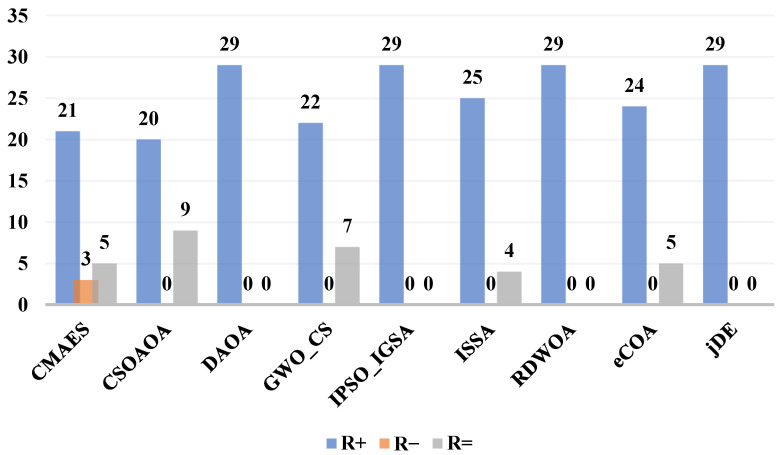
Wilcoxon test results of ACIVY versus other advanced algorithms using CEC2017.

On the other hand, the Wilcoxon rank-sum test results shown in Figure 7 provide comprehensive pairwise validation of ACIVY’s dominance, revealing overwhelming statistical superiority against all competing algorithms. ACIVY demonstrates complete dominance against CSOAOA, DAOA, GWO_CS, ISSA, RDWOA, eCOA, and jDE with R+ values of 20, 29, 22, 29, 25, 29, 24, and 29, respectively, while maintaining zero R− values across all comparisons. Against CMAES, ACIVY shows superior performance on 21 functions with only 3 inferior performances and 5 ties, representing a 7:1 win-to-loss ratio. The R= values indicate occasional statistical ties with CMAES (5), CSOAOA (9), GWO_CS (7), RDWOA (4), and jDE (5), but these represent performance equivalence rather than losses. The absence of R− values against most algorithms demonstrates ACIVY’s remarkable consistency in outperforming advanced state-of-the-art methods across diverse optimization scenarios, establishing unequivocal statistical evidence of its algorithmic superiority in high-dimensional optimization contexts.

### 4.2. ACIVY for Engineering Problems

To assess the real-world effectiveness of the proposed ACIVY algorithm, it is applied to a suite of well-known engineering design problems. These include optimization tasks such as the design of a welded beam, speed reducer problem, tension/compression spring, tubular column, robotic gripper, speed reducer, and three-bar truss design problem. Each problem includes a set of design constraints, which are handled using a static penalty mechanism as outlined in Equation (22) [82].

In this penalty-based formulation, the modified objective function is expressed as ζ(z), where f(z) is the original objective. The penalties are incorporated using the terms $o_{j}$ and $l_{i}$, which are positive constants. The expressions $U_{j}(z)$ and $T_{i}(z)$ correspond to inequality and equality constraints, respectively. To control the severity of penalties, exponents α and β are introduced, each set to either 1 or 2, depending on the constraint handling requirements.

(22) $$\zeta (z)=f(z)\pm \left[\sum_{i=1}^{m}\;\;l_{i}\cdot max{\left(0,T_{i}(z)\right)}^{\alpha }+\sum_{j=1}^{n}\;\;o_{j}\cdot {\left|U_{j}(z)\right|}^{\beta }\right]$$

Each engineering problem is solved under identical algorithmic settings to ensure fair performance comparison. The experimental setup includes a fixed population size of 50, a total of 500 iterations, and 30 separate runs for each problem to obtain statistically meaningful results. The main mathematical modelling of these problems is presented in Appendix A.

#### 4.2.1. The Tension–Compression Spring Design Problem

The tension–compression spring design (TCSD) task can be described as follows [83]: given the total weight of the spring, find the minimum possible weight subject to a number of engineering-based constraints. The proposed optimization problem can be characterized by three primary variables, including the thickness of the wires x1, the mean diameter of the coils x2, and the number of active coils x3. These variables need to be finely tuned to meet design specifications regarding stresses limits, deformation limits, and mechanical reliability. The resulting design should be able to resist tension and compression forces, the material must be used efficiently, and the spring life should be maximized. It is used as a benchmark to evaluate the effectiveness and constraint-handling capability of state-of-the-art metaheuristic algorithms. The mathematical model of this problem can be found in Appendix A. Table 3 shows the results of running the performance evaluation assignment uses the tension–compression spring design problem to illustrate that ACIVY has an outstanding optimization ability. ACIVY provides the best mean fitness value of 0.012665233 with an extremely small standard deviation of 8.69008 × 10^−17^, which implies flawless convergence uniformity throughout the 30 independent runs. This performance is impressively better than all the competing algorithms, with the nearest rival TTAO having a mean fitness of 0.012705868 and a much larger standard deviation of 2.27993 × 10^−5^. The good performance of the algorithm is also revealed by the fact that it found the same best and worst fitness, which has never been seen before in terms of reliability and robustness in handling constraints and global optimization. The optimal design parameters obtained by ACIVY and other comparative algorithms, as shown in Table 4, reveal a well-balanced engineering solution with a wire diameter ($x_{1}$) of 0.0516890607, a mean coil diameter ($x_{2}$) of 0.356717730, and a number of active coils ($x_{3}$) of 11.2889664, resulting in the best fitness value of 0.0126652328. Compared to other algorithms, ACIVY’s parameter configuration represents a practical and manufacturable design that effectively balances material usage with structural performance requirements.

#### 4.2.2. Weight Minimization of a Speed Reducer

The speed reducer design problem represents a well-known case in mechanical engineering optimization, where the primary goal is to minimize the total mass of a gearbox assembly without compromising its mechanical performance or its compatibility with connected components such as the engine and propeller system [23]. Reducing the system’s weight not only improves energy efficiency but also contributes to better operational performance and reduced load on supporting structures. The seven design variables that correspond to this problem represent an important geometry or mechanical behavior feature of the reducer. The optimization algorithm is subject to numerous design constraints, which include material strength, stress tolerances, and geometric feasibility. The mathematical model of this problem can be found in Appendix A. The optimization results for the speed reducer design problem, presented in Table 5, demonstrate ACIVY’s exceptional performance in handling complex multi-dimensional constrained optimization. ACIVY achieves an optimal mean fitness value of 2713.031561 with an extraordinarily small standard deviation of 0.000149, indicating remarkable consistency and reliability across all independent runs. This performance significantly surpasses competing algorithms, with the second-best performer, TTAO, achieving a nearly identical mean fitness of 2713.034866 but with a substantially higher standard deviation of 0.009914. The algorithm’s superiority is further emphasized when compared to other methods like SWO (2845.973890) and SCA (2857.789669), which show a considerably worse performance with high variance, demonstrating ACIVY’s robust capability in navigating the complex constraint landscape of this seven-dimensional optimization problem.

Moreover, the optimal design parameters obtained by ACIVY, as detailed in Table 6, reveal a well-engineered solution with a face width ($x_{1}$) of 2.80700516, module of teeth ($x_{2}$) of 0.70000000, number of teeth on pinion ($x_{3}$) of 17.00000000, shaft lengths ($x_{4},x_{5}$) of 7.30000000 each, and bearing diameters ($x_{6},x_{7}$) of 3.35054095 and 5.28651792, respectively, resulting in the best fitness value of 2713.03156062. Notably, ACIVY and MFO achieve close parameter configurations, indicating convergence to the true global optimum, while other algorithms like SWO and WOA show significant deviations from optimal values, particularly in the variables $x_{1}$, $x_{2}$, and $x_{3}$. The parameter analysis reveals that ACIVY consistently identifies design configurations that satisfy all mechanical constraints while minimizing weight, with several variables converging to boundary values ($x_{2}=0.7,x_{3}=17.0,x_{4}=x_{5}=7.3$), suggesting these represent critical design limits for optimal performance in speed reducer applications.

#### 4.2.3. Welded Beam Design Problem

This optimization problem addresses the cost-effective manufacturing of a welded beam structure [84]. The objective is to minimize the fabrication cost by adjusting four geometric parameters: weld thickness $x_{1}$, beam height $x_{2}$, beam length $x_{3}$, and beam flange thickness $x_{4}$. The mathematical model of this problem can be found in Appendix A. The optimization results for the welded beam design problem, presented in Table 7, showcase ACIVY’s outstanding performance in minimizing fabrication costs while satisfying complex structural constraints. ACIVY achieves an optimal mean fitness value of 1.67021773 with an exceptionally small standard deviation of 0.00000006, demonstrating near-perfect convergence consistency across all runs. This performance substantially outperforms the competing algorithms, with TTAO achieving a very close mean fitness of 1.67021777 but with a significantly higher standard deviation of 0.00485478. The algorithm’s superiority becomes more pronounced when compared to other methods such as SWO (3.13525785), TSA (2.09364637), and WOA (2.41742026), which exhibit a considerably worse performance with high variance, indicating ACIVY’s exceptional capability in handling the nonlinear constraints and complex design space of this structural optimization problem.

The optimal design parameters obtained by ACIVY, as detailed in Table 8, reveal a highly efficient structural configuration with a weld thickness ($x_{1}$) of 0.1988323072, beam height ($x_{2}$) of 3.3373652986, beam length ($x_{3}$) of 9.1920243225, and beam flange thickness ($x_{4}$) of 0.1988323072, resulting in the best fitness value of 1.67021772. The parameter analysis shows that ACIVY, TTAO, and several other algorithms converge to close values, confirming the global optimum identification. Notably, ACIVY achieves perfect symmetry with $x_{1}=x_{4}$, indicating an optimal design balance between weld and flange thicknesses. In contrast, algorithms like SWO and TSA show significant parameter deviations, particularly in the beam height ($x_{2}$) values of 5.3649639962 and 3.4191767660, respectively, resulting in substantially higher costs and demonstrating ACIVY’s superior constraint handling and convergence precision in structural design optimization applications.

#### 4.2.4. Three-Bar Truss Design Problem

This design optimization scenario focuses on a traditional three-bar truss structure exposed to applied external forces [85]. The primary aim is to reduce the overall use of material—quantified by the structure’s weight—while guaranteeing that stress levels across all members remain within permissible limits when subjected to loading. The decision variables in this model are $x_{1}$ and $x_{2}$, representing the cross-sectional areas of the horizontal and angled (diagonal) elements of the truss, respectively. The mathematical model of this problem can be found in Appendix A. The optimization outcomes of the three-bar truss design problem in Table 9 show that ACIVY is very effective in minimizing the weight of the structure subject to the satisfaction of stress constraints. ACIVY provides a higher mean fitness value of 98.9054388 with an incredibly small standard deviation of 0.0000002, which implies excellent consistency and stability throughout the optimization runs. This compares favorably to most of the competing algorithms with a few having a slightly higher mean fitness value of 98.9675309 but almost a similar standard deviation of 0.0000003. The advantage of using the algorithm can be observed compared to approaches such as SWO (98.5799219 +/− 2.5137111) and SCA (99.7838562 +/− 1.5109708), which showed much higher variance and less guaranteed convergence, showing the strong performance of ACIVY in searching the nonlinear constraint space of this structural optimization problem.

The design parameters yielded by ACIVY, tabulated in Table 10, indicate the optimum design with the structure showing an efficient configuration with cross-sectional areas $x_{1}$ = 0.270343428 and $x_{2}$= 0.225028620, having the best fitness value of 98.905430382. Interestingly, ACIVY, DOA, and IVY appear to converge to the same parameter values of both design variables, indicating convergence to the actual global optimum region. The parameter analysis indicates that the ACIVY has the best performance in terms of the cross-sectional area of the horizontal member ($x_{1}$) and diagonal member ($x_{2}$), where the horizontal member needs around 20% more cross-sectional area to accommodate the structural loads efficiently.

### 4.3. ACIVY for UAV Path Planning Optimization

In order to evaluate the practical relevance of the suggested ACIVY algorithm, its performance is considered in the area of unmanned aerial vehicle (UAV) navigation planning. Based on the fundamental assessments presented in Section 4.1 and Section 4.2, in which ACIVY demonstrated stable superiority over traditional and state-of-the-art MAs on the CEC2017 and CEC2022 benchmarks, this section applies it to the UAV path planning problem. The benchmark functions are renowned for having complicated traits such as high dimensionality, nonlinearity, and discontinuities that serve as good analogues of the problems posed by UAV trajectory design. Accordingly, the ACIVY algorithm will be tested in a simulated setting with circular areas of threats, as seen in real-life applications in defense, surveillance, and autonomous aerospace missions.

The UAV path planning problem can be formulated as follows: given a certain starting point and destination, find a trajectory that is optimal in some sense, with the constraints of operating in an environment filled with potential obstacles and operational constraints [86]. Such risks might be in the form of terrain obstacles, enemy detection areas, or restricted airspaces. Moreover, the UAV has to observe physical limitations such as fuel capacity, dynamics, and maneuverability limits, like the turn radius and maximum climb rate. The operating environment is often idealized to a 2D or 3D grid with geometric obstacles, frequently assumed to be circles or polygons.

**Figure 8:**
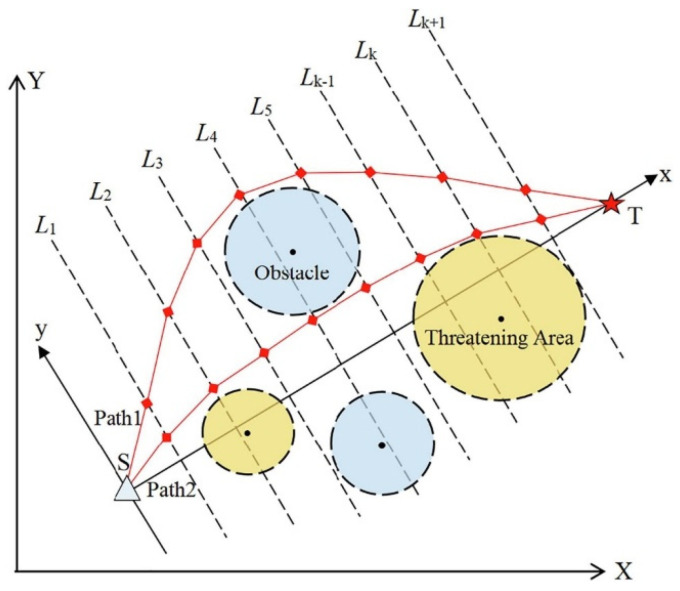
UAV path planning diagram [86].

In this configuration, the object has to drive around doughnut-shaped areas, which are interpreted as regions of high risk, e.g., radar detection fields or dangerous terrain. Figure 8 depicts that the UAV is initially at a known starting point S and has to reach the desired destination T. The direct line between S and T is split into K segments equally, which brings flexibility to the routing procedure. Also, the lines $L_{1}$, $L_{2}$,…, $L_{K}$, $L_{K+1}$ perpendicular to the segmentation points are constructed. The lines each delineate a plane of potential discrete waypoints amongst which one optimum point may be chosen.

By connecting one point on each of these perpendicular segments in consecutive fashion, a closed and valid flight path is created that meets mission requirements and avoids any prescribed threat areas or restricted areas. Using this structure, a flexible, but constrained, optimization problem is enabled to precisely capture the nature of the operational needs of autonomous UAV systems. The complete set of navigation points is rigorously stated as

(23) $$C=\left\{S,L_{1}(x(1),y(1)),L_{2}(x(2),y(2)),\ldots ,L_{K}(x(K),y(K)),L_{K+1}(x(D),y(D)),T\right\}$$

Such a discrete representation enables the ACIVY algorithm to search through a wide range of feasible paths and converge to one that achieves the minimization of exposure and fuel consumption and the feasibility of the route, considering environmental and vehicle-specific constraints. A coordinate transformation is used to ease the calculations and to match the optimization direction with the geometry of the problem. The transformation that moves the segment ST into the new x-axis is

(24) $$\left[\begin{array}{l} x^{*}(k)\\ y^{*}(k) \end{array}\right]=\left[\begin{array}{cc} cos\theta & sin\theta \\ -sin\theta & cos\theta \end{array}\right]\left[\begin{array}{l} x(k)-x_{S}\\ y(k)-y_{S} \end{array}\right]$$

where

(25) $$\theta =arcsin\frac{y_{T}-y_{S}}{\left|ST\right|}$$

(26) $$x(k)=\frac{\left|ST\right|}{K+1}\times k.k=1,2,\ldots ,K$$

Here, θ represents the angular deviation between the original x-axis and the segment ST, allowing the trajectory generation to occur along a normalized reference frame. The fitness function used in this case is defined in terms of cost and fuel as shown in Appendix A.

To thoroughly evaluate the proposed ACIVY algorithm in the context of UAV navigation, simulation experiments are carried out across different environmental configurations, each populated with circular threat zones. These test cases differ in threat zone density, spatial positioning, and risk magnitude, simulating a wide range of practical mission scenarios. The UAV begins its flight at position (2,2) and is required to reach the target location at (3299,3299), modeling a long-distance mission through potentially adversarial or restricted airspace.

The experimental configuration is the same in all the simulations. We limit each optimization process to 1000 iterations, and the size of the population is 30 solutions per generation. The problem space is characterized by 10 dimensions, which is equal to the number of intermediate decisions that constitute the path of the UAV. Each algorithm is run independently 30 times to make the statistical results reliable and consider the stochasticity of MA. The result of each run is stored, and such key performance indicators as the best, worst, average, and standard deviation of the final cost are calculated to compare.

The performance of ACIVY is compared to some well-known metaheuristic algorithms, which are the original Butterfly Optimization Algorithm (BOA) [87], Crayfish Optimization Algorithm (CFish) [65], Human Memory Optimization (HMO) [68], Tunicate Swam Algorithm (TSA) [88], Arithmetic Optimization Algorithm (AOA) [62], Coati Optimization Algorithm (COA) [64], and the original IVYA algorithm. The mentioned algorithms are well known to be successful in solving continuous and constrained optimization problems with nonlinear objective functions.

The strength and correctness of ACIVY are approved in three test cases that represent various spatial distributions of circular threat zones. The scenarios, as concluded in Table 11, differ in the concentration of obstacles, size, and intensity, providing a wide range of assessments. These findings demonstrate that ACIVY is not only an efficient method to deal with nonlinear and high-dimensional trajectory optimization but also it is stable in various runs.

As observed in Table 12, the results of the performance evaluation of Case 1 show that ACIVY is exceptionally competent in resolving the UAV path planning optimization problem in the first threat scenario setup. ACIVY provides the best performance in terms of all the statistical metrics because it has the best fitness value of 2502.12372, a mean fitness of 2502.32598, and the standard deviation is extremely low, 0.264660329. This extreme performance is properly several times faster than any of the competing algorithms, with the nearest rival BOA having a best fitness of 2504.41698 and a mean of 2506.60476, making the performance difference between this and the other algorithms. The advantage of the algorithm performance is even more significant in comparison to such methods as CFish (mean: 2536.58402 +/− 27.02260666) and TSA (mean: 2532.1528 +/− 16.26598301) that have a significantly higher cost and variance.

The statistical comparison shows a remarkable consistency and reliability of ACIVY in UAV mission planning, as its standard deviation is extremely low compared to all the competitors. Though such algorithms as IVY (2508.98792 +/− 2.318207734) and HMO (2510.85416 +/− 2.372573147) demonstrate a certain consistency in performance, they remain far behind in accuracy when compared to ACIVY. Such consistency is especially important in real-world UAVs where the reliability of missions and predictability of performance are top priorities, and as seen in the case of ACIVY with a relatively small gap between the best (2502.12372) and worst (2502.8327) performance values, shows the robustness of its algorithm in dynamic path planning tasks.

**Figure 9:**
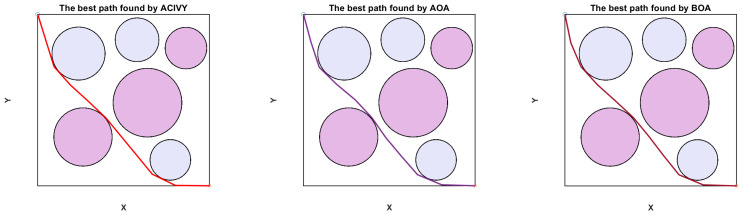

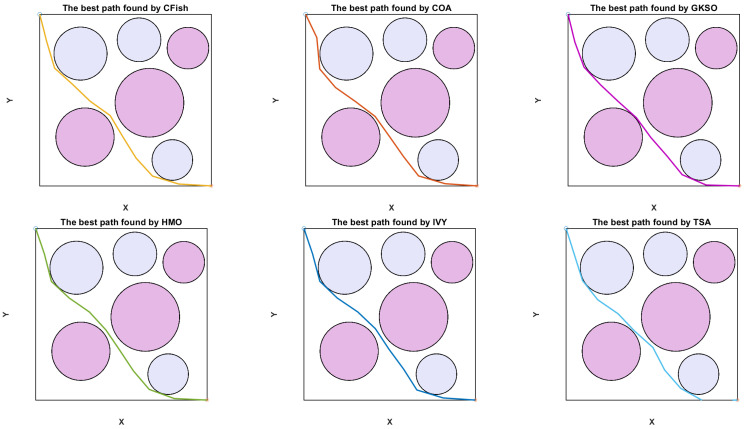
Path planning results for Case 1 using various algorithms.

The visual optimal paths computed by each algorithm in Case 1, presented in Figure 9, show the different high-level strategies of threat avoidance and trajectory optimization. The trajectory of ACIVY shows better spatial intelligence, as it follows the best balance between directness and safety, performing smooth curved movements to optimally go around the six circular threat areas while keeping the distance and the threat levels minimal. The trajectory covered by the algorithm has a well-chosen waypoint that results in smooth arcs around the high-risk regions, especially visible in how it avoids the large threat area in the center and the concentration of threats in the upper-right quarter. Conversely, other algorithms, such as TSA and COA, are more conservative and exhibit extreme curvature that leads to higher fuel consumption, whereas BOA and HMO tend to produce angles that are too sharp and could lead to UAV maneuverability limitations.

The comparative analysis shows a wide disparity in the quality and feasibility of paths found by the competing algorithms. The path of ACIVY shows a constant clearance margin to all threat boundaries without sacrificing the smoothness of the flight path, which implies a better constraint handling and multi-objective optimization ability. The algorithm is able to find the optimal corridor along the complex threat landscape without making unnecessary detours, and without entering dangerously close to high-intensity threat areas. Other algorithms, such as CFish and GKSO, exhibit a poor path planning performance, either by going too near the high-risk regions or by making unnecessary long diversions, which leads to an increase in the cost of the mission.

**Figure 10:**
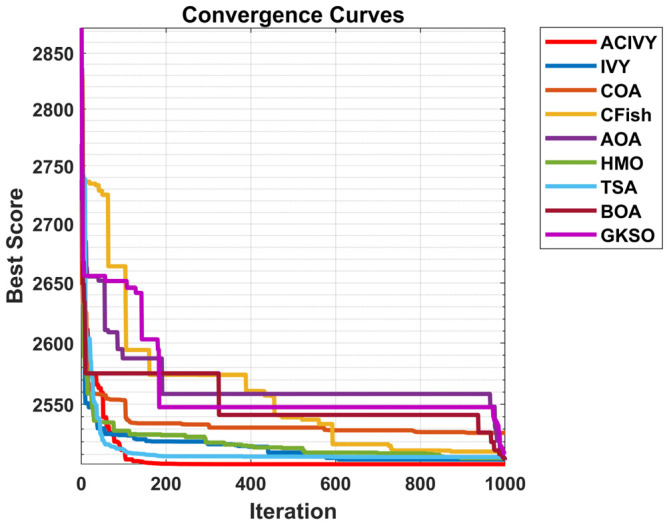
Convergence curve for Case 1.

The convergence curve of Case 1, shown in Figure 10, indicates the outstanding optimization dynamics and the excellent convergence properties of ACIVY within the UAV path planning application field. ACIVY shows the most aggressive and effective early-stage convergence behavior, falling quickly out of an initial cost of about 2900 to near-optimal costs of about 2500 in the first 100 iterations. Such a fast convergence implies that the algorithm has strong exploration abilities and effective search space exploration, which makes it quick to find and exploit good areas of the solution space. The algorithm shows consistent progress in optimization, and in the end, gives the minimum final cost compared to any other competitor. Comparatively, the GKSO and BOA algorithms exhibit slower rates of initial convergence, whereas CFish and TSA exhibit less reliable improvement trends with temporary periodical stagnation.

The convergence stability plot indicates the excellent consistency and stable optimization pattern of ACIVY during the 1000 iterations. Following the fast initial progress, ACIVY continues with a smooth and steady refinement process, which is evidence of well-balanced exploration and exploitation dynamics that avoid premature convergence, at the same time guaranteeing a constantly improving solution. The convergence curve of the algorithm has very few variations with no observable plateaus, indicating strong local optima escape mechanisms and good global search abilities. Such consistent convergence behavior is in stark contrast to other algorithms, such as COA and HMO, that show erratic convergence behavior with many plateau periods and little progress in later iterations. The enviable convergence traits of ACIVY are in direct proportion to its statistical performance indices, thus validating the role played by the increased bio-inspired strategies in the algorithm to convert the high convergence rate into a steady state of optimal solution quality in the testing UAV path planning environments.

Case 2 performance analysis presented in Table 13 shows that ACIVY remains a clear leader in dealing with more complex threats, with the best fitness value of 2374.11064 and an incredible consistency demonstrated by the standard deviation of just 0.234912478. This constitutes a strong performance improvement over its nearest rival, BOA, which has a best fitness of 2375.47342 and a mean of 2376.87129, showing that ACIVY optimizes better in this harder threat landscape. The performance of the algorithm is further accentuated when compared to algorithms suffering significantly during the higher threat density, especially CFish (mean: 2470.77789 +/− 65.84699354) and TSA (mean: 2451.45497 +/− 32.77127957), which demonstrate extreme performance degradation and high variance in the Case 2 complex scenario.

**Figure 11:**
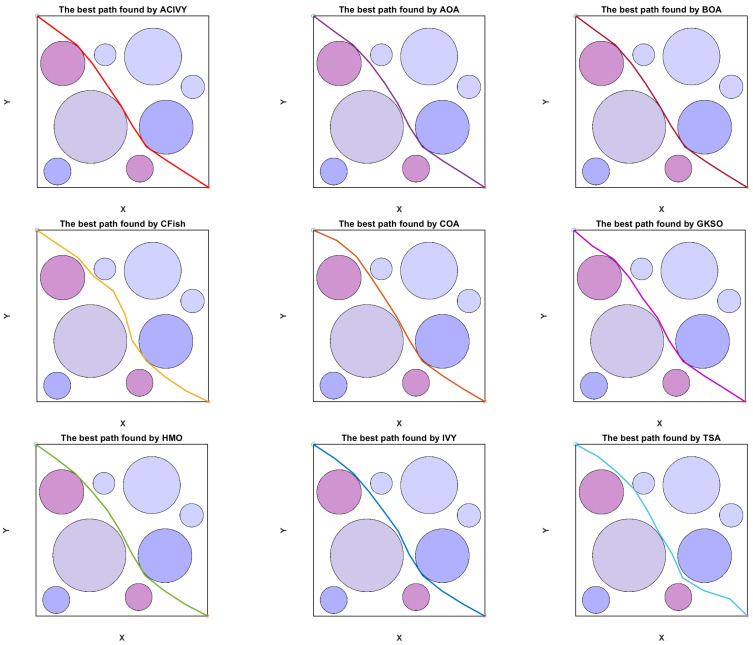
Path planning results for Case 2 using various algorithms.

On the other hand, Figure 11 shows the visualization of Case 2, which indicates that ACIVY outperformed its spatial reasoning skills in the more challenging threat landscape consisting of eight distributed circular areas with different intensities. The trajectory of ACIVY is characterized by remarkable strategic analysis, showing the best path across the thick threat field, keeping an almost straight line, but carefully bypassing all high-risk sectors. The track of the algorithm illustrates highly advanced waypoint selection to develop a smooth executable path with minimal offset to the straight-line distance, efficiently balancing the two competing goals of threat avoidance and fuel optimization. It is worth noting that the ACIVY route includes going between the central large threats with safe clearance margins, demonstrating the high level of constraint processing in the complex multi-threat case.

The comparison demonstrates that the difference in the quality of paths found by the competing algorithms vastly widens in the presence of greater environmental complexity. TSA shows the most cautious case of over-curvature that significantly adds mission distance and fuel burn, and CFish displays improper planning of an unjustifiably winding track that generates numerous direction alterations. Other algorithms, such as AOA, BOA, and COA, perform moderately well on relatively straight-line paths but do not have the accuracy of the best corridor discovery exhibited by ACIVY.

**Figure 12:**
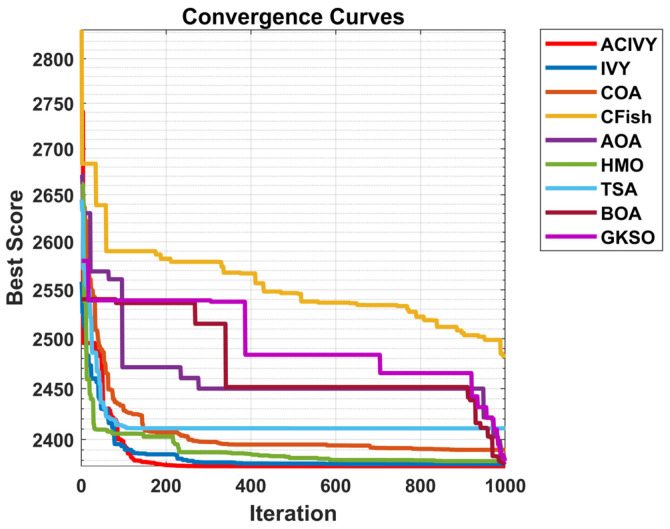
Convergence curve for Case 2.

The convergence pattern in Figure 12 shows that ACIVY in Case 2 is adjusted remarkably well to higher complexity in the environment, and the optimization dynamics were faster than in Case 1. ACIVY also converges to near-optimal values of around 2380 after only 50 iterations, demonstrating that it explores the more crowded threat environment of Case 2 more efficiently. The algorithm retains its typical smooth convergence profile with no oscillations or early stagnation and ends up with the final minimum cost among all the competitors. This improved convergence rate in Case 2 indicates that the improved strategies in ACIVY are specifically suitable for the complex multi-threat environment where the traditional algorithms fail due to higher computational requirements and the complexity of the solution space.

Finally, the Case 3 performance evaluation at Table 14 shows a much complex optimization terrain with ten threat zones, but ACIVY stays on the leadership track with the best fitness value of 2433.65864, indicating a high-quality solution even in the complex environment. Nevertheless, Case 3 demonstrates some curious performance patterns according to which the standard deviation (24.2325059) in ACIVY is higher than in the former cases, which might be interpreted as the increased complexity of the given threat constellation. Our nearest rival, COA, shows a competitive average performance of 2444.96932 with very low variance (4.0979329), whereas IVY is consistently ranked second with a best fitness of 2441.37365 and acceptable reliability (16.9353954 standard deviation). The performance of such algorithms as CFish (2478.17905 +/− 6.22824561) and AOA (2478.61011 +/− 9.35102086) is worse than in previous instances, which proves the higher level of optimization challenge provided by the dense threat configuration of Case 3.

Statistical analysis yields the peculiar challenge profile of Case 3, in which the ten-threat setting provides a more intricate optimization terrain and challenges the robustness and adaptiveness of the algorithms. Although ACIVY achieves the optimal individual solution, the higher variance implies that this specific threat configuration contains local optima issues, which sometimes impact the consistency of convergence. In this case, COA shows an excellent stability with the least standard deviation compared to other algorithms, which means that it is able to handle constraints well, even though it finds suboptimal solutions. The difference between the best (2433.65864) and mean (2444.2745) performance of ACIVY demonstrates that though the algorithm can find optimal corridors in the maze of threatening agents, convergence to the global optimum becomes more difficult in this crowded environment, which shows the implied complexity of ten-threat navigation scenarios with regards to UAV path planning problems.

**Figure 13:**
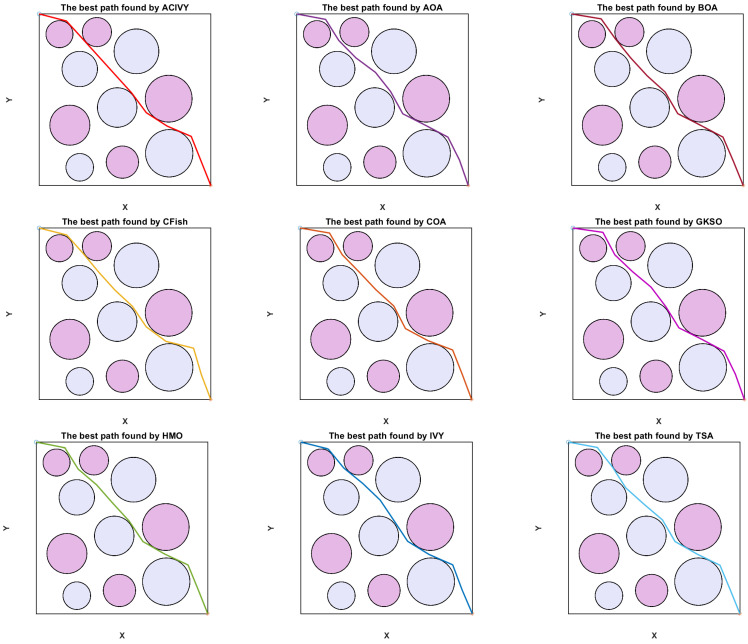
Path planning results for Case 3 using various algorithms.

Figure 13 presents the path routes for various algorithms in Case 3, which indicates the tremendous complexity caused by the ten-threat array, with ACIVY displaying highly advanced spatial navigation through the densest threat array in the experiment. The path of ACIVY demonstrates a high-level optimization of waypoints, as it found a viable corridor that demands careful maneuvering around several tightly spaced threats, especially in its lower part, where the algorithm has to thread the needle between the close group of four threats, still keeping safe clearance distances. The route exhibits a degree of tactical flexibility in that the curvature is controlled and varies dynamically according to the threat proximity, giving a smooth but dynamic route that balances the needs of directness with the safety constraints. Such an advanced navigation feature is the main difference between ACIVY and its competitors, TSA and IVY, which are characterized by less aggressive styles but with too many deviations that prolong the mission time and fuel usage.

Moreover, to address the challenge of real-world applicability and demonstrate the robustness of ACIVY in handling complex geometric configurations beyond simple circular obstacles, an additional experimental scenario was designed involving 3D path planning through an oil plant industrial environment [89,90]. This complex scenario represents a significant advancement from the previous circular threat zone experiments, incorporating realistic industrial structures, including pipelines, storage tanks, processing units, and multi-level platforms that UAVs commonly encounter in industrial inspection and monitoring missions. The oil plant environment presents a particularly challenging navigation landscape due to its intricate 3D geometry, varying obstacle heights, narrow corridors between equipment, and the critical safety requirements inherent in petrochemical facilities where collision avoidance is paramount.

**Figure 14:**
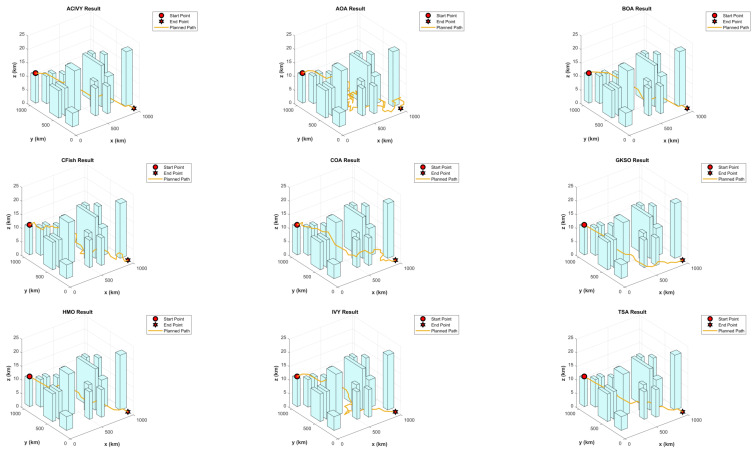
Map of shortest obtained path of 3D oil plant’s path planning.

The statistical performance analysis presented in Table 15 demonstrates ACIVY’s exceptional capability in navigating this complex 3D industrial environment, achieving the best fitness value of 31.97736 with a remarkably low standard deviation of 0.11129, indicating superior consistency and reliability across multiple optimization runs. ACIVY’s performance is closely matched by BOA, which achieves an identical best fitness of 31.97736 but with higher variance (0.36918), while HMO ranks third with a competitive performance (32.07374 ± 0.25926). The algorithm significantly outperforms other competitors, with IVY showing a substantially degraded performance (44.39475 ± 4.76428) and CFish struggling considerably in this complex environment (46.96253 ± 1.43415). The visual path planning results illustrated in Figure 14 clearly demonstrate the superior spatial reasoning capabilities of ACIVY, which successfully identifies optimal corridors through the dense industrial infrastructure while maintaining safe clearance margins from all structural components. The trajectory generated by ACIVY exhibits remarkable smoothness and directness, efficiently navigating between multi-level structures and avoiding collision with complex geometric shapes, including cylindrical tanks, rectangular processing units, and interconnected piping systems. In contrast to the smooth, efficient trajectory generated by ACIVY, competing algorithms exhibit various degrees of suboptimal behavior, including unnecessary detours, inefficient altitude changes, and potentially hazardous proximity to industrial equipment. The path visualization reveals that while some algorithms like BOA and HMO produce relatively acceptable routes, others, such as CFish and IVY generate highly irregular trajectories with excessive maneuvering that would be impractical for real UAV operations, thereby validating the enhanced optimization strategies integrated into ACIVY for handling real-world complex geometric constraints in critical industrial applications.

### 4.4. Results Discussion

The comprehensive experimental validation demonstrates ACIVY’s consistent superiority across diverse optimization domains. ACIVY achieved first-place rankings on both the CEC2022 (average rank 1.25) and CEC2017 (average rank 1.41) benchmark suites, delivered perfect convergence on four engineering design problems, and demonstrated an optimal UAV path planning performance across simple circular threats and complex 3D industrial environments, establishing its broad applicability and reliability.

The experimental results reveal fundamental weaknesses in competing algorithms that explain their poor performance on specific function types. Algorithms like AOA and COA exhibit severe limitations on multimodal and composite functions due to premature convergence and population diversity loss, where their search mechanisms cause early clustering around local optima, effectively reducing global exploration to local refinement. For instance, AOA’s performance on CEC2022 composite functions (F9–F12) shows fitness values worse than ACIVY, while CFish demonstrates trajectory irregularities in UAV path planning due to its temperature-based search strategy that creates oscillatory behavior preventing convergence in constrained environments. TSA suffers from swarm movement trapping in complex hybrid function landscapes, lacking sophisticated escape mechanisms when populations become confined in fitness valleys created by multi-characteristic function combinations.

ACIVY systematically addresses these limitations through its three integrated enhancement strategies, each targeting specific algorithmic weaknesses observed in competitors. The CCS strategy solves the population diversity crisis plaguing algorithms like AOA by introducing structured horizontal and vertical crossover operations that maintain genetic spread while preserving solution quality, enabling successful navigation of multimodal landscapes where competitors prematurely converge. The LT strategy provides the directional guidance deficiency observed in algorithms like TSA and CFish through its memory-based navigation system that combines historical position information with adaptive repulsion from poor regions, explaining ACIVY’s smooth UAV trajectories versus the erratic paths generated by competing methods. The TGAM strategy resolves the exploration–exploitation transition problems evident in traditional algorithms through its dynamic selection pool mechanism, ensuring gradual phase transitions that prevent both premature convergence and excessive exploration, particularly effective on hybrid and composite functions requiring balanced global–local search capabilities.

The experimental results reveal important insights regarding algorithmic scalability and robustness across different problem dimensions and characteristics. ACIVY demonstrates remarkable consistency between CEC2017 (50D) and CEC2022 (20D) performance, maintaining its top ranking across both benchmark suites despite the dimensional differences and varying function complexities. This scalability stems from the dimension-independent nature of the three enhancement strategies, which operate on relative population relationships rather than absolute dimensional coordinates. In contrast, algorithms like CMA-ES and jDE show significant performance degradation on higher-dimensional problems, indicating scalability limitations in their core mathematical frameworks.

The robustness analysis across engineering applications and UAV path planning scenarios reveals ACIVY’s exceptional constraint handling capabilities compared to competing algorithms. While traditional optimization methods often struggle with feasibility maintenance in constrained problems, ACIVY’s integrated strategies naturally preserve constraint satisfaction through their guided search mechanisms. The LT strategy’s repulsion component inherently avoids infeasible regions, while the CCS strategy’s structured recombination tends to generate feasible offspring from feasible parents. This explains ACIVY’s perfect convergence on highly constrained problems like tension–compression spring design, where competing algorithms exhibit high variance due to constraint violations and subsequent penalty applications.

## 5. Conclusions and Future Works

To enhance the optimization capabilities of the basic ivy-inspired algorithm, this paper introduces an advanced variant named ACIVY through the integration of three strategic enhancement mechanisms: the CC strategy, the LT strategy, and the TGAM strategy. By incorporating formulations from complementary algorithmic approaches that demonstrate significant impact on overall optimization performance, ACIVY delivers superior solutions to complex optimization challenges while maintaining computational efficiency without an excessive complexity overhead. Firstly, the CC strategy facilitates both horizontal information exchange between different ivy individuals and vertical recombination within individual solutions, thereby broadening the exploration capacity of the algorithm across the solution landscape while enhancing population diversity. Secondly, recognizing that ivy solutions may become trapped in suboptimal regions during the optimization process, the LT strategy incorporates directional memory and repulsion mechanisms from poorly performing areas, introducing more effective escape strategies into the algorithmic framework. Through the integration of positional memory and worst-solution avoidance, the algorithm significantly improves its capability to navigate away from local optima traps. Finally, by adaptively selecting solutions from top-performing candidates with dynamically adjusted selection pools, the TGAM enhances the algorithm’s exploitation capabilities through a smooth transition mechanism that begins with broad exploration and gradually focuses on elite regions. After selecting classical, recently developed, and enhanced intelligent algorithms as comparative baselines, the comprehensive performance of ACIVY is evaluated and analyzed on the CEC2017 benchmark suite in 50 dimensions and the CEC2022 benchmark suite at maximum dimensionality. Following the demonstration of ACIVY’s strong competitiveness in both moderate- and high-dimensional optimization scenarios through rigorous benchmark evaluations, ACIVY is further applied to optimize engineering design problems, structural optimization challenges, and UAV path planning systems, thereby validating its practical applicability.

Although ACIVY consistently identifies superior solutions across the majority of test functions and optimization problems examined in this research, its performance exhibits room for improvement in certain specialized cases. Taking the convergence behavior on specific multimodal functions as examples, while ACIVY successfully locates global optimal regions, the presence of numerous deceptive local optima occasionally delays the algorithm’s convergence to the precise global optimum position. The algorithm demonstrates excellent exploration capabilities but may require additional refinement mechanisms for faster exploitation in highly complex landscapes. Therefore, future research directions consider incorporating advanced strategies from other successful algorithmic paradigms or developing more sophisticated enhancement mechanisms to further optimize the ivy-inspired framework. For instance, convergence acceleration could be achieved through chaotic mapping techniques that improve initial population distribution across the solution space, while the overall algorithmic performance could be evaluated more comprehensively using additional diverse benchmark suites and increasingly challenging optimization scenarios. Moreover, hybrid approaches combining ACIVY with local search operators or gradient-based refinement techniques could enhance convergence precision in continuous optimization domains. Additionally, extending ACIVY to address other practical optimization challenges presents promising research opportunities, including applications to multi-objective optimization problems, parameter estimation tasks, image segmentation applications, feature selection methodologies, path planning optimization, constrained portfolio optimization, and various engineering design optimization problems. Furthermore, developing specialized variants of ACIVY for discrete optimization problems, dynamic optimization environments, and large-scale optimization scenarios represents valuable directions for future algorithmic development and practical implementation across diverse computational optimization domains.

## Figures and Tables

**Table 1:** Various parameter settings.

Algorithm	Parameter Value
IVY	beta1=[1,1.5),GV=[0,1]
CFish	$c_{3}=3,\mu =25,\sigma =3$
SWO	TR=0.3,CR=0.2
HHO	$E_{0}$ changes from −1 to 1
AOA	α=5;μ=0.5
SHO	$r_{1}=0$
GJO	$c_{1}varies\;from\;1\;to\;2$
WOA	k=1,q=[−1,1]
HMO	r=0.1,δ=1
CMA-ES	*σ* = 0.5, *μ* = *λ*/2
CSOAOA	μ=0.499,a=5
GWO_CS	a: Linear reduction from 2 to 0
RDWOA	a1=[2,0],a2=[−2,−1],s=0,b=1
ISSA	PP=0.2,ST=0.8

**Table 2:** Experimental results using CEC2022 for ACIVY and other competitors.

F		ACIVY	IVY	CFish	SWO	HHO	AOA	SHO	COA	GJO	WOA	HMO
F1	AVG	300.367	2.04 × 10^4^	2.97 × 10^4^	2.89 × 10^4^	1.07 × 10^4^	3.44 × 10^4^	1.63 × 10^4^	4.67 × 10^4^	1.67 × 10^4^	2.52 × 10^4^	2.88 × 10^4^
	STD	0.354	7.12 × 10^3^	1.15 × 10^4^	7.87 × 10^3^	4.57 × 10^3^	1.15 × 10^4^	5.67 × 10^3^	1.67 × 10^4^	5.58 × 10^3^	9.65 × 10^3^	5.50 × 10^3^
	RAN	1.000	5.000	9.000	8.000	2.000	10.000	3.000	11.000	4.000	6.000	7.000
F2	AVG	454.503	461.213	480.930	817.469	500.719	2313.817	734.730	2739.419	601.308	602.597	527.389
	STD	14.464	16.524	32.738	130.026	44.203	691.341	162.768	903.166	83.583	58.302	56.421
	RAN	1.000	2.000	3.000	9.000	4.000	10.000	8.000	11.000	6.000	7.000	5.000
F3	AVG	600.000	612.237	632.378	646.757	660.884	663.864	635.415	681.533	624.441	668.814	650.756
	STD	0.000	17.810	19.190	9.677	7.843	9.821	7.072	8.641	10.493	11.722	14.072
	RAN	1.000	2.000	4.000	6.000	8.000	9.000	5.000	11.000	3.000	10.000	7.000
F4	AVG	861.190	869.064	885.626	966.783	890.779	951.796	901.316	976.023	898.858	926.999	918.394
	STD	16.220	19.579	11.856	21.108	10.207	19.520	18.889	13.490	19.060	34.035	27.567
	RAN	1.000	2.000	3.000	10.000	4.000	9.000	6.000	11.000	5.000	8.000	7.000
F5	AVG	1402.843	2330.722	2497.418	3557.366	2748.341	2905.607	2294.742	3488.791	1891.702	4313.402	2844.974
	STD	585.628	217.876	473.138	925.472	347.055	503.066	243.251	274.136	384.528	2023.432	879.971
	RAN	1.000	4.000	5.000	10.000	6.000	8.000	3.000	9.000	2.000	11.000	7.000
F6	AVG	5.61 × 10^3^	9.60 × 10^7^	5.16 × 10^3^	6.48 × 10^7^	1.44 × 10^5^	9.13 × 10^8^	1.60 × 10^7^	2.36 × 10^9^	3.31 × 10^7^	6.84 × 10^5^	1.19 × 10^5^
	STD	5.07 × 10^3^	5.26 × 10^8^	4.76 × 10^3^	7.01 × 10^7^	7.85 × 10^4^	7.76 × 10^8^	3.47 × 10^7^	1.00 × 10^9^	7.73 × 10^7^	8.81 × 10^5^	9.68 × 10^4^
	RAN	2.000	9.000	1.000	8.000	4.000	10.000	6.000	11.000	7.000	5.000	3.000
F7	AVG	2056.696	2137.905	2143.352	2191.550	2176.636	2237.169	2115.099	2214.742	2138.678	2218.054	2193.709
	STD	41.771	73.703	110.297	47.021	61.173	78.547	28.259	35.238	64.246	73.738	73.155
	RAN	1.000	3.000	5.000	7.000	6.000	11.000	2.000	9.000	4.000	10.000	8.000
F8	AVG	2260.457	2353.952	2285.581	2295.767	2280.644	2526.417	2262.081	2422.545	2250.708	2281.428	2260.005
	STD	62.807	105.482	70.404	48.043	85.281	201.288	53.566	130.614	41.786	55.849	46.855
	RAN	3.000	9.000	7.000	8.000	5.000	11.000	4.000	10.000	1.000	6.000	2.000
F9	AVG	2480.811	2482.172	2480.838	2622.093	2506.411	3106.709	2578.705	3367.518	2585.750	2590.748	2586.161
	STD	0.054	2.563	0.067	53.994	17.804	220.027	43.869	325.034	40.048	67.705	50.669
	RAN	1.000	3.000	2.000	9.000	4.000	10.000	5.000	11.000	6.000	8.000	7.000
F10	AVG	2656.389	3797.680	3659.552	3332.965	3970.456	5776.169	3196.159	6221.128	3286.555	4981.016	4395.223
	STD	186.648	997.377	1073.671	1379.774	554.202	758.705	753.281	1277.091	1259.521	995.349	730.315
	RAN	1.000	6.000	5.000	4.000	7.000	10.000	2.000	11.000	3.000	9.000	8.000
F11	AVG	2900.000	3136.262	2969.089	5028.901	3052.952	8278.623	5421.058	8638.962	4664.856	3694.190	3282.689
	STD	90.972	339.959	68.482	629.180	152.609	895.911	719.050	511.625	488.771	981.596	196.656
	RAN	1.000	4.000	2.000	8.000	3.000	10.000	9.000	11.000	7.000	6.000	5.000
F12	AVG	2959.470	3058.215	2982.821	3245.125	3190.484	3842.580	3146.542	3629.908	3029.246	3078.993	3131.113
	STD	20.201	79.767	28.596	78.894	216.126	224.550	93.148	321.741	61.201	97.770	88.022
	RAN	1	4	2	9	8	11	7	10	3	5	6
Average rank		1.25	4.42	4.00	8.00	5.08	9.92	5.00	10.50	4.25	7.58	6.00
Final rank		1	4	2	9	6	10	5	11	3	8	7

**Table 3:** Statistical results for different algorithms on the tension–compression spring problem.

Algorithm	Mean Fit	Std Fit	Best Fit	Worst Fit
ACIVY	0.012665233	8.69008 × 10^−17^	0.012665233	0.012665233
TTAO	0.012705868	2.27993 × 10^−5^	0.012666401	0.012719054
GJO	0.012882226	0.000208677	0.012710990	0.013208584
SWO	0.022105307	0.009278603	0.016836736	0.038546846
GWO	0.012921274	0.000317903	0.012732738	0.013472578
TSA	0.013659016	0.000643385	0.013044764	0.014578979
HMO	0.012944524	0.000308650	0.012713229	0.013464599
DOA	0.012968371	0.004739516	0.012732000	0.013300526
MFO	0.013249026	0.000733529	0.012719054	0.014520913
IVY	0.012677291	2.51722 × 10^−5^	0.012665426	0.012722310
WOA	0.013460376	0.000636634	0.012719972	0.014140797
SCA	0.013555008	0.000748813	0.013197411	0.014894123

**Table 4:** The obtained parameters of the tension–compression spring problem.

	Dim 1	Dim 2	Dim 3	Best Fit
ACIVY	0.0516890607	0.356717730	11.2889664	0.0126652328
TTAO	0.0514580023	0.351181440	11.6212147	0.0126664012
GJO	0.0509222999	0.338016513	12.5019173	0.0127109896
SWO	0.0627991451	0.651678189	4.5511458	0.0168367364
GWO	0.0500000000	0.317309583	14.0508707	0.0127327377
TSA	0.0563848789	0.480563648	6.5380758	0.0130447640
HMO	0.0507533863	0.334470451	12.7559805	0.0127132292
DOA	0.0500000000	0.250000000	2.0000000	0.0127320000
MFO	0.0500000000	0.317425416	14.0277697	0.0127190537
IVY	0.0517781489	0.358864373	11.1642590	0.0126654260
WOA	0.0534272677	0.399993082	9.1405789	0.0127199724
SCA	0.0500000000	0.310527328	15.0000000	0.0131974114

**Table 5:** Statistical results for different algorithms on the speed reducer engineering problem.

Algorithm	Mean Fit	Std Fit	Best Fit	Worst Fit
ACIVY	2713.031561	0.000149	2713.031561	2713.031561
TTAO	2713.034866	0.009914	2713.035205	2713.042789
GJO	2735.384399	8.548108	2725.878386	2746.498767
SWO	2845.973890	41.715280	2806.655741	2891.039379
GWO	2725.800166	4.407300	2720.250429	2730.271725
TSA	2800.056985	46.984929	2756.035440	2860.974057
HMO	2739.296737	6.343501	2730.452570	2744.773583
DOA	2768.397508	54.553211	2722.344357	2847.153116
MFO	2715.176574	4.098385	2713.031561	2722.487329
IVY	2713.557535	0.413185	2713.070425	2714.120243
WOA	2738.435550	16.338263	2726.378238	2765.157234
SCA	2857.789669	23.748792	2835.813799	2891.331329

**Table 6:** The obtained parameters of the speed reducer engineering problem.

	Dim 1	Dim 2	Dim 3	Dim 4	Dim 5	Dim 6	Dim 7	Best Fit
ACIVY	2.80700516	0.70000000	17.00000000	7.30000000	7.30000000	3.35054095	5.28651792	2713.03156062
TTAO	2.80700356	0.70000020	17.00000000	7.30000001	7.30000975	3.35054095	5.28649199	2713.035205
GJO	2.80607645	0.70000000	17.00000000	7.56960632	7.78284951	3.35275349	5.28598152	2725.87838558
SWO	2.65266131	0.71953145	17.04453823	7.97213600	7.35020823	3.36943322	5.39007453	2806.65574124
GWO	2.76807571	0.70288441	17.04464763	7.36275450	7.45019823	3.35774583	5.28694648	2720.25042935
TSA	2.67316462	0.71723221	17.00183862	7.63179595	7.66042048	3.43969287	5.28657991	2756.03543982
HMO	2.68109611	0.71620421	17.00000000	7.48615530	7.40724349	3.35440028	5.29444027	2730.45256971
DOA	2.80700276	0.70000000	17.00000000	8.28681295	7.30226839	3.35250442	5.28657790	2722.34435720
MFO	2.80700516	0.70000000	17.00000000	7.30000000	7.30000000	3.35054095	5.28651792	2713.03156062
IVY	2.80619714	0.70006230	17.00093432	7.30000003	7.30003775	3.35054271	5.28651760	2713.07042547
WOA	2.60000000	0.70000000	17.67379500	7.97175151	7.30000000	3.35124425	5.28737074	2726.37823820
SCA	2.60000000	0.73039166	17.00000000	7.30000000	7.30000000	3.57771708	5.34142453	2835.81379866

**Table 7:** Statistical results for different algorithms on the welded beam engineering problem.

Algorithm	Mean Fit	Std Fit	Best Fit	Worst Fit
ACIVY	1.67021773	0.00000006	1.67021772	1.67021773
TTAO	1.67021777	0.00485478	1.67021773	1.67021788
GJO	1.67798447	0.00324986	1.67325770	1.68185252
SWO	3.13525785	0.79817987	2.27932543	4.40770380
GWO	1.67886408	0.01209177	1.67299271	1.70048410
TSA	2.09364637	0.19643593	1.88066831	2.38323220
HMO	1.67998635	0.00617658	1.67357101	1.68791939
DOA	2.26711217	0.97478894	1.67023578	3.93704622
MFO	1.72615602	0.08646018	1.67022080	1.86781042
IVY	1.67022211	0.00000762	1.67021790	1.67023571
WOA	2.41742026	0.58495909	1.81790132	3.07151163
SCA	1.81225898	0.03912541	1.77144095	1.86058556

**Table 8:** The obtained parameters of the welded beam engineering problem.

	Dim 1	Dim 2	Dim 3	Dim 4	Best Fit
ACIVY	0.1988323072	3.3373652986	9.1920243225	0.1988323072	1.6702177262
TTAO	0.1988323072	3.3373652987	9.1920243224	0.1988323072	1.6702177263
GJO	0.1982526539	3.3586717806	9.1917854011	0.1989794701	1.6732577009
SWO	0.1280094865	5.3649639962	9.6759577509	0.2420750504	2.2793254337
GWO	0.1987215007	3.3436391192	9.2001619898	0.1989315640	1.6729927131
TSA	0.2104588003	3.4191767660	8.2171713211	0.2488081578	1.8806683078
HMO	0.1983028134	3.3439689315	9.2083754891	0.1989036555	1.6735710101
DOA	0.1988155247	3.3376885536	9.1920250809	0.1988323066	1.6702357823
MFO	0.1988331504	3.3373546714	9.1920048328	0.1988331504	1.6702207962
IVY	0.1988321651	3.3373680138	9.1920242240	0.1988323128	1.6702179019
WOA	0.1878659650	3.7762076700	9.9998646301	0.1953538917	1.8179013235
SCA	0.2117136082	3.6377456963	8.9803992061	0.2088247004	1.7714409502

**Table 9:** Statistical results for different algorithms on the three-bar truss engineering problem.

Algorithm	Mean Fit	Std Fit	Best Fit	Worst Fit
ACIVY	98.9054388	0.0000002	98.9054304	98.9675308
TTAO	98.9662565	0.0075418	98.9550531	98.9734130
GJO	99.1529703	0.2083844	98.8422735	99.4189758
SWO	98.5799219	2.5137111	97.1184147	103.0499027
GWO	99.0557764	0.0668505	98.9418371	99.1122830
TSA	98.9137920	0.2377723	98.5064150	99.0928317
HMO	98.9675306	0.1411411	98.7945967	99.1439281
DOA	98.9675309	0.0000003	98.9675305	98.9675313
MFO	98.9137900	0.2824333	98.4259553	99.1618534
IVY	98.9675309	0.0000003	98.9675305	98.9675312
WOA	99.0438681	0.2430006	98.8166210	99.4368257
SCA	99.7838562	1.5109708	97.4146163	101.5515781

**Table 10:** The obtained parameters of the three-bar truss engineering problem.

	Dim 1	Dim 2	Best Fit
ACIVY	0.270343428	0.225028620	98.905430382
TTAO	0.270320606	0.224968398	98.955053112
GJO	0.270119043	0.224410708	98.842273526
SWO	0.265029424	0.221567734	97.118414667
GWO	0.270296677	0.224903918	98.941837122
TSA	0.269499010	0.222805841	98.506415002
HMO	0.270028611	0.224189719	98.794596700
DOA	0.270343428	0.225028620	98.967530485
MFO	0.269351281	0.222419083	98.425955322
IVY	0.270343428	0.225028620	98.967530477
WOA	0.270067493	0.224299989	98.816621031
SCA	0.266744585	0.219678543	97.414616303

**Table 11:** Description of threats in the 3 cases.

Case Number	Threat Center		Threat Radius	Threat Grade
Case 1	755	785	510	8
	2360	870	560	2
	490	1910	420	1
	1700	2110	660	7
	2800	2550	390	9
	650	2850	400	5
Case 2	900	500	415	7
	2140	1050	720	3
	2990	390	260	1
	740	1300	210	3
	780	2220	550	3
	1360	2990	230	5
	2130	2480	510	8
	2930	1970	260	2
Case 3	390	390	260	8
	1060	780	340	7
	2140	590	385	2
	2950	780	265	1
	350	1110	280	4
	1800	1500	380	7
	2850	1600	310	5
	720	1870	430	2
	1630	2490	450	5
	2680	2500	455	8

**Table 12:** Statistical metrics for Case 1.

Algorithm	Best	Worst	Mean	Std
ACIVY	2502.12372	2502.8327	2502.32598	0.264660329
IVY	2505.79595	2513.50757	2508.98792	2.318207734
COA	2516.90609	2542.77181	2533.14868	8.997407103
CFish	2511.78067	2592.83355	2536.58402	27.02260666
AOA	2505.28289	2531.34269	2512.19472	7.920026613
HMO	2506.68865	2514.37586	2510.85416	2.372573147
TSA	2507.69716	2558.49118	2532.1528	16.26598301
BOA	2504.41698	2509.10282	2506.60476	1.547602521
GKSO	2504.95517	2526.95066	2514.0311	7.370667795

**Table 13:** Statistical metrics for Case 2.

Algorithm	Best	Worst	Mean	Std
ACIVY	2374.11064	2374.79358	2374.34822	0.234912478
IVY	2375.93232	2389.05456	2380.26256	4.259265757
COA	2390.04828	2401.79987	2394.19953	3.92472551
CFish	2386.69344	2557.27769	2470.77789	65.84699354
AOA	2377.57035	2393.93684	2384.58996	5.195902807
HMO	2376.37967	2384.59586	2380.72904	2.864422909
TSA	2410.7254	2502.91243	2451.45497	32.77127957
BOA	2375.47342	2378.75988	2376.87129	1.098992628
GKSO	2377.91747	2408.44774	2384.58935	9.221217975

**Table 14:** Statistical metrics for Case 3.

Algorithm	Best	Worst	Mean	Std
ACIVY	2433.65864	2493.71135	2444.2745	24.2325059
IVY	2441.37365	2490.76837	2463.78362	16.9353954
COA	2440.67175	2452.77517	2444.96932	4.0979329
CFish	2469.49883	2484.92828	2478.17905	6.22824561
AOA	2466.56975	2489.08886	2478.61011	9.35102086
HMO	2477.23665	2495.6077	2484.72095	6.97409205
TSA	2445.3967	2457.69732	2451.42206	4.83704743
BOA	2444.01371	2480.29167	2459.94161	14.6152532
GKSO	2450.4893	2481.85431	2467.87812	12.1957948

**Table 15:** Statistical metrics for complex 3D path planning.

Algorithm	Best	Worst	Mean	Std
ACIVY	31.97736	32.17011	32.10586	0.11129
IVY	38.99854	48.01972	44.39475	4.76428
COA	37.58433	46.94453	42.62214	4.72093
CFish	45.67658	48.50913	46.96253	1.43415
AOA	52.06742	50.06742	52.06742	1.74 × 10^−14^
HMO	32.07374	32.56315	32.36789	0.25926
TSA	32.65953	33.83110	33.08217	0.65037
BOA	31.97736	32.65953	32.23688	0.36918
GKSO	33.48795	43.04843	37.40570	5.00823

**Figure A1:**
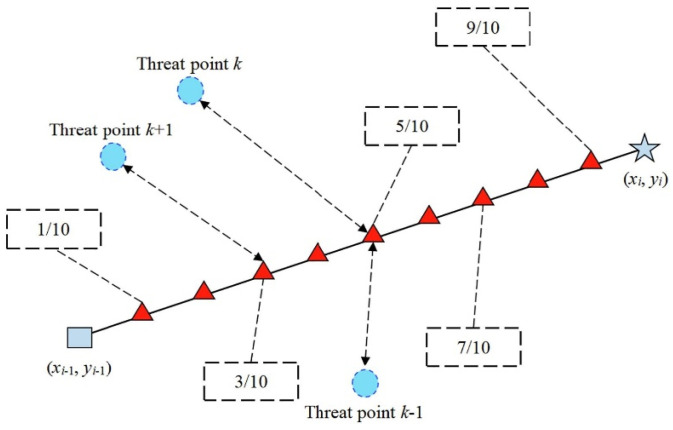
Threat cost computation.

## Data Availability

The data presented in this study are available on request from the corresponding author.

## Appendix A

**Table A1:** Experimental results using CEC2017 for ACIVY and advanced competitors, 50D.

F		ACIVY	eCOA	CMAES	DAOA	CSOAOA	GWO_CS	RDWOA	jDE	ISSA	IPSO_IGSA
F1	AVG	6296.553	1.00 × 10^10^	2.03 × 10^10^	2.83 × 10^11^	1.77 × 10^8^	1.18 × 10^10^	1.85 × 10^10^	1.48 × 10^11^	1.76 × 10^8^	3.75 × 10^10^
	STD	5477.757	2.29 × 10^9^	3.15 × 10^10^	9.47 × 10^9^	8.05 × 10^7^	4.53 × 10^9^	5.24 × 10^9^	4.81 × 10^10^	8.66 × 10^7^	9.55 × 10^9^
	RAN	1.000	4.000	7.000	10.000	3.000	5.000	6.000	9.000	2.000	8.000
F3	AVG	37,241.988	1.22 × 10^5^	3.81 × 10^5^	5.26 × 10^6^	1.17 × 10^5^	1.15 × 10^5^	1.73 × 10^5^	3.20 × 10^5^	2.15 × 10^5^	1.73 × 10^5^
	STD	8359.405	2.08 × 10^4^	4.63 × 10^4^	1.10 × 10^7^	1.84 × 10^4^	1.42 × 10^4^	2.65 × 10^4^	2.97 × 10^4^	4.96 × 10^4^	1.39 × 10^4^
	RAN	1.000	4.000	9.000	10.000	3.000	2.000	5.000	8.000	7.000	6.000
F4	AVG	506.774	1605.778	8973.258	1.04 × 10^5^	643.383	2312.396	4.30 × 10^3^	5.25 × 10^4^	699.060	8605.554
	STD	57.144	435.158	1806.282	1.87 × 10^4^	54.336	820.052	1.65 × 10^3^	5.77 × 10^3^	111.890	1919.301
	RAN	1.000	4.000	8.000	10.000	2.000	5.000	6.000	9.000	3.000	7.000
F5	AVG	758.024	829.114	543.371	1718.310	845.294	811.883	1032.024	1425.927	886.634	862.306
	STD	53.530	48.528	7.753	77.012	16.869	31.781	42.402	62.299	46.148	93.840
	RAN	2.000	4.000	1.000	10.000	5.000	3.000	8.000	9.000	7.000	6.000
F6	AVG	602.144	655.855	629.382	754.527	636.195	632.831	684.760	718.346	667.237	675.034
	STD	3.740	9.689	32.310	6.934	8.250	8.159	5.137	7.008	6.542	17.765
	RAN	1.000	5.000	2.000	10.000	4.000	3.000	8.000	9.000	6.000	7.000
F7	AVG	953.746	1531.379	918.635	6209.619	1400.290	1148.987	1721.889	4419.215	1729.234	1765.704
	STD	62.602	156.631	94.533	526.048	141.217	61.772	70.062	546.234	82.015	205.980
	RAN	2.000	5.000	1.000	10.000	4.000	3.000	6.000	9.000	7.000	8.000
F8	AVG	1048.075	1121.699	854.876	1966.989	1147.244	1104.868	1279.610	1703.818	1196.065	1214.746
	STD	41.928	17.861	12.470	97.508	27.802	46.632	44.501	140.777	15.577	139.949
	RAN	2.000	4.000	1.000	10.000	5.000	3.000	8.000	9.000	6.000	7.000
F9	AVG	7113.715	11,300.780	900.076	1.04 × 10^5^	1.26 × 10^4^	1.87 × 10^4^	2.33 × 10^4^	5.86 × 10^4^	1.43 × 10^4^	1.45 × 10^4^
	STD	2850.799	4084.546	0.185	1.42 × 10^4^	1.11 × 10^3^	5.30 × 10^3^	3.47 × 10^3^	5.07 × 10^3^	1.12 × 10^3^	4.91 × 10^3^
	RAN	2.000	3.000	1.000	10.000	4.000	7.000	8.000	9.000	5.000	6.000
F10	AVG	7.89 × 10^3^	7.82 × 10^3^	1.45 × 10^4^	1.69 × 10^4^	6.98 × 10^3^	7.72 × 10^3^	1.27 × 10^4^	1.48 × 10^4^	8.88 × 10^3^	1.49 × 10^4^
	STD	1529.99^3^	1090.771	358.649	667.760	116.768	970.002	1697.274	1712.986	751.117	766.314
	RAN	4.000	3.000	7.000	10.000	1.000	2.000	6.000	8.000	5.000	9.000
F11	AVG	1282.802	2036.636	5.99 × 10^4^	8.52 × 10^4^	2.94 × 10^3^	6.41 × 10^3^	6.22 × 10^3^	3.33 × 10^4^	2.20 × 10^3^	1.54 × 10^4^
	STD	44.416	354.813	1.10 × 10^4^	1.68 × 10^4^	8.14 × 10^2^	2.49 × 10^3^	1.98 × 10^3^	6.49 × 10^3^	2.44 × 10^2^	3.08 × 10^3^
	RAN	1.000	2.000	9.000	10.000	4.000	6.000	5.000	8.000	3.000	7.000
F12	AVG	5.89 × 10^6^	3.83 × 10^8^	1.75 × 10^10^	1.41 × 10^11^	3.94 × 10^7^	3.93 × 10^9^	4.35 × 10^9^	5.89 × 10^10^	6.04 × 10^7^	8.99 × 10^9^
	STD	4.07 × 10^6^	1.19 × 10^8^	4.73 × 10^9^	2.45 × 10^10^	2.19 × 10^7^	2.02 × 10^9^	1.92 × 10^9^	1.16 × 10^10^	3.83 × 10^7^	3.73 × 10^9^
	RAN	1.000	4.000	8.000	10.000	2.000	5.000	6.000	9.000	3.000	7.000
F13	AVG	9.39 × 10^3^	4.25 × 10^4^	9.30 × 10^9^	8.00 × 10^10^	5.28 × 10^5^	4.38 × 10^7^	5.24 × 10^8^	2.63 × 10^10^	7.37 × 10^4^	2.79 × 10^8^
	STD	9.73 × 10^3^	2.56 × 10^3^	2.09 × 10^9^	1.95 × 10^10^	3.93 × 10^5^	6.22 × 10^7^	2.79 × 10^8^	7.10 × 10^9^	4.68 × 10^4^	6.17 × 10^8^
	RAN	1.000	2.000	8.000	10.000	4.000	5.000	7.000	9.000	3.000	6.000
F14	AVG	1.78 × 10^5^	8.61 × 10^5^	2.14 × 10^7^	3.46 × 10^8^	2.09 × 10^6^	1.17 × 10^6^	3.57 × 10^6^	2.07 × 10^7^	1.79 × 10^6^	6.89 × 10^6^
	STD	9.30 × 10^4^	6.91 × 10^5^	1.54 × 10^7^	2.18 × 10^8^	2.73 × 10^6^	1.53 × 10^6^	1.06 × 10^6^	8.45 × 10^6^	1.18 × 10^6^	4.88 × 10^6^
	RAN	1.000	2.000	9.000	10.000	5.000	3.000	6.000	8.000	4.000	7.000
F15	AVG	1.34 × 10^4^	2.92 × 10^4^	1.55 × 10^9^	2.59 × 10^10^	1.18 × 10^5^	4.96 × 10^6^	8.06 × 10^7^	5.06 × 10^9^	2.48 × 10^4^	7.78 × 10^6^
	STD	6.71 × 10^3^	4.66 × 10^3^	6.34 × 10^8^	1.47 × 10^10^	1.34 × 10^5^	8.45 × 10^6^	7.44 × 10^7^	5.10 × 10^9^	9.46 × 10^3^	1.90 × 10^7^
	RAN	1.000	3.000	8.000	10.000	4.000	5.000	7.000	9.000	2.000	6.000
F16	AVG	3049.852	3947.981	6792.996	15,592.660	3500.837	3189.571	5926.594	9055.936	4292.301	5766.571
	STD	379.670	264.659	380.797	2072.610	393.166	383.071	642.244	497.937	409.176	1225.147
	RAN	1.000	4.000	8.000	10.000	3.000	2.000	7.000	9.000	5.000	6.000
F17	AVG	2927.292	3573.512	2.88 × 10^3^	9.41 × 10^5^	3.61 × 10^3^	2.82 × 10^3^	4143.599	15,027.332	3806.724	3858.856
	STD	356.456	379.507	3.40 × 10^2^	6.56 × 10^5^	3.48 × 10^2^	1.41 × 10^2^	289.005	6635.532	406.534	329.599
	RAN	3.000	4.000	2.000	10.000	5.000	1.000	8.000	9.000	6.000	7.000
F18	AVG	1.21 × 10^6^	3.41 × 10^6^	1.09 × 10^8^	9.49 × 10^8^	2.53 × 10^6^	8.30 × 10^6^	3.64 × 10^7^	9.61 × 10^7^	3.46 × 10^6^	1.27 × 10^7^
	STD	5.67 × 10^5^	2.87 × 10^6^	4.26 × 10^7^	7.53 × 10^8^	7.62 × 10^5^	9.89 × 10^6^	2.92 × 10^7^	4.97 × 10^7^	2.45 × 10^6^	7.96 × 10^6^
	RAN	1.000	3.000	9.000	10.000	2.000	5.000	7.000	8.000	4.000	6.000
F19	AVG	2.44 × 10^4^	3.67 × 10^5^	9.31 × 10^8^	1.42 × 10^10^	1.08 × 10^5^	3.13 × 10^6^	8.07 × 10^6^	2.63 × 10^9^	1.94 × 10^4^	6.23 × 10^5^
	STD	1.49 × 10^4^	8.83 × 10^4^	6.75 × 10^8^	5.74 × 10^9^	1.95 × 10^5^	4.42 × 10^6^	8.86 × 10^6^	1.93 × 10^9^	1.37 × 10^4^	5.30 × 10^5^
	RAN	2.000	4.000	8.000	10.000	3.000	6.000	7.000	9.000	1.000	5.000
F20	AVG	2759.503	3208.329	3659.770	5438.454	2978.333	3112.379	3618.170	4400.183	3505.598	3959.967
	STD	241.676	259.246	207.275	80.018	190.226	552.003	181.385	159.281	329.363	263.273
	RAN	1.000	4.000	7.000	10.000	2.000	3.000	6.000	9.000	5.000	8.000
F21	AVG	2524.357	2649.598	2665.898	3612.063	2633.605	2541.018	2977.799	3267.381	2908.815	2756.079
	STD	41.279	37.738	257.329	78.436	42.783	32.381	99.312	70.780	77.585	137.769
	RAN	1.000	4.000	5.000	10.000	3.000	2.000	8.000	9.000	7.000	6.000
F22	AVG	9001.506	9942.091	1.64 × 10^4^	1.83 × 10^4^	9.43 × 10^3^	8.95 × 10^3^	1.45 × 10^4^	1.68 × 10^4^	1.15 × 10^4^	1.62 × 10^4^
	STD	1219.994	1040.759	2.42 × 10^2^	5.78 × 10^2^	8.07 × 10^2^	2.09 × 10^3^	1.92 × 10^3^	8.92 × 10^2^	2.06 × 10^3^	1.02 × 10^3^
	RAN	2.000	4.000	8.000	10.000	3.000	1.000	6.000	9.000	5.000	7.000
F23	AVG	2939.509	3139.797	3447.945	5085.375	3083.851	3044.229	3662.255	4334.166	3458.461	3833.051
	STD	49.593	44.276	43.871	355.448	50.229	65.281	231.890	126.546	166.907	193.502
	RAN	1.000	4.000	5.000	10.000	3.000	2.000	7.000	9.000	6.000	8.000
F24	AVG	3108.387	3279.692	3547.729	6067.681	3593.713	3305.331	3737.815	4586.738	3731.748	4068.991
	STD	31.500	88.503	42.933	227.167	119.822	98.674	106.266	133.212	97.253	222.115
	RAN	1.000	2.000	4.000	10.000	5.000	3.000	7.000	9.000	6.000	8.000
F25	AVG	3077.383	3910.330	3445.823	6.69 × 10^4^	3.22 × 10^3^	4.00 × 10^3^	5.02 × 10^3^	2.70 × 10^4^	3236.897	6717.841
	STD	26.042	164.000	225.584	7.00 × 10^3^	2.24 × 10^1^	3.79 × 10^2^	3.68 × 10^2^	2.74 × 10^3^	42.430	800.381
	RAN	1.000	5.000	4.000	10.000	2.000	6.000	7.000	9.000	3.000	8.000
F26	AVG	2900.045	1.21 × 10^4^	1.19 × 10^4^	2.99 × 10^4^	1.11 × 10^4^	7993.782	12,587.718	20,291.813	8350.629	1.38 × 10^4^
	STD	0.028	9.15 × 10^2^	5.28 × 10^2^	4.47 × 10^3^	1.37 × 10^3^	2518.307	860.370	1401.049	3175.798	1.22 × 10^3^
	RAN	1.000	6.000	5.000	10.000	4.000	2.000	7.000	9.000	3.000	8.000
F27	AVG	3532.013	3805.025	3929.404	8946.756	3617.956	3818.542	4820.190	6126.356	3662.657	5578.163
	STD	96.030	159.127	145.788	1120.766	102.432	121.287	128.783	557.167	219.147	465.615
	RAN	1.000	4.000	6.000	10.000	2.000	5.000	7.000	9.000	3.000	8.000
F28	AVG	3336.037	4580.758	1.00 × 10^4^	2.60 × 10^4^	3.53 × 10^3^	4692.495	5669.511	14,520.241	3581.775	7687.407
	STD	44.597	355.999	3.49 × 10^2^	2.23 × 10^3^	4.52 × 10^1^	309.998	374.417	4186.913	51.875	826.931
	RAN	1.000	4.000	8.000	10.000	2.000	5.000	6.000	9.000	3.000	7.000
F29	AVG	4252.657	6487.372	1.49 × 10^4^	2.47 × 10^6^	4.51 × 10^3^	5.03 × 10^3^	9.35 × 10^3^	1.89 × 10^4^	5852.306	9587.065
	STD	282.579	629.473	2.58 × 10^3^	1.75 × 10^6^	3.69 × 10^2^	4.39 × 10^2^	2.04 × 10^3^	4.55 × 10^3^	701.319	4778.510
	RAN	1.000	5.000	8.000	10.000	2.000	3.000	6.000	9.000	4.000	7.000
F30	AVG	1.15 × 10^6^	1.33 × 10^8^	2.59 × 10^9^	1.91 × 10^10^	1.13 × 10^6^	1.51 × 10^8^	2.68 × 10^8^	5.13 × 10^9^	4.21 × 10^6^	2.89 × 10^8^
	STD	2.18 × 10^5^	1.45 × 10^7^	7.19 × 10^8^	7.31 × 10^9^	1.16 × 10^5^	4.38 × 10^7^	1.28 × 10^8^	1.33 × 10^9^	1.38 × 10^6^	1.17 × 10^8^
	RAN	2	4	8	10	1	5	6	9	3	7
Average rank		1.41	3.79	6.00	10.00	3.17	3.72	6.69	8.83	4.38	7.00
Final rank		1	4	6	10	2	3	7	9	5	8

The mathematical model of tension–compression problem can be described as follows:

Consider	
	$\begin{array}{c} x=[x_{1}x_{2}x_{3}]=[dDN] \end{array}$
Objective function:	
	$\begin{array}{c} f\left(x\right)=\left(x_{3}+2\right)\times x_{2}\times x_{1}^{2} \end{array}$
Subject to	
	$\begin{array}{ccc} g_{1}\left(x\right) & = & 1-\frac{x_{3}\times x_{2}^{3}}{71785\times x_{1}^{4}}\leq 0\\ g_{2}\left(x\right) & = & \frac{4\times x_{2}^{2}-x_{1}\times x_{2}}{12566\times x_{1}^{4}}+\frac{1}{5108\times x_{1}^{2}}-1\leq 0\\ g_{3}\left(x\right) & = & 1-\frac{140.45\times x_{1}}{x_{2}^{2}\times x_{3}}\leq 0\\ g_{4}\left(x\right) & = & \frac{x_{1}+x_{2}}{1.5}-1\leq 0 \end{array}$
Boundaries:	
	$\begin{array}{ccc} 0.05 & \leq & x_{1}\leq 2.0\\ 0.25 & \leq & x_{2}\leq 1.3\\ 2.0 & \leq & x_{3}\leq 15.0 \end{array}$

The mathematical model of speed-reducer problem can be described as follows:

Objective function:

$$\begin{array}{rr} minf(x)= & 0.7854x_{1}x_{2}^{2}\left(3.3333x_{3}^{2}+14.9334x_{3}-43.0934\right)-1.508x_{1}\left(x_{6}^{3}+x_{7}^{2}\right)\\ & \;+7.4777\left(x_{6}^{3}+x_{7}^{3}\right)+0.7854\left(x_{4}x_{6}^{2}+x_{5}x_{7}^{2}\right) \end{array}$$

Constraint condition:

$$g_{1}(x)=\frac{27}{x_{1}x_{2}^{2}x_{3}}-1\leq 0$$

$$\begin{array}{c} g_{2}(x)=\frac{397.5}{x_{1}x_{2}^{2}x_{3}}-1\leq 0\\ g_{3}(x)=\frac{1.93x_{5}^{3}}{x_{1}x_{6}^{4}x_{3}}-1\leq 0\\ g_{4}(x)=\frac{1.93x_{5}^{3}}{x_{2}x_{7}^{4}x_{3}}-1\leq 0\\ g_{5}(x)=\frac{{\left[{\left(745\left(x_{4}/x_{2}x_{3}\right)\right)}^{2}+16.9\times 10^{6}\right]}^{1/2}}{110x_{6}^{3}}-1\leq 0\\ g_{6}(x)=\frac{{\left[{\left(745\left(x_{5}/x_{2}x_{3}\right)\right)}^{2}+157.5\times 10^{6}\right]}^{1/2}}{85x_{7}^{3}}-1\leq 0\\ g_{7}(x)=\frac{x_{2}x_{3}}{40}-1\leq 0\\ g_{8}(x)=\frac{5x_{2}}{x_{1}}-1\leq 0 \end{array}$$

$$\begin{array}{c} g_{9}(x)=\frac{5x_{1}}{12x_{2}}-1\leq 0\\ g_{10}(x)=\frac{1.5x_{6}+1.9}{x_{4}}-1\leq 0\\ g_{11}(x)=\frac{1.1x_{7}+1.9}{x_{5}}-1\leq 0 \end{array}$$

Value range:

$$\begin{array}{c} 2.6\leq x_{1}\leq 3.6\\ 0.7\leq x_{2}\leq 0.8\\ 17\leq x_{3}\leq 28\\ 7.3\leq x_{4}\\ x_{5}\leq 8.3\\ 2.9\leq x_{6}\leq 3.9 \end{array}$$

$$5.0\leq x_{7}\leq 5.5$$

The mathematical model of welded beam problem can be defined as follows:

$$minf(x)=1.10471x_{1}^{2}x_{2}+0.04811x_{3}^{2}x_{4}\left(14+x_{2}\right)$$

Subject to the following constraints:

$$\begin{array}{r} g_{1}(x)=H(x)-H_{max}\leq 0\\ g_{2}(x)=\sigma (x)-M_{max}\leq 0\\ g_{3}(x)=\delta (x)-\delta_{max}\leq 0\\ g_{4}(x)=x_{1}-x_{4}\leq 0\\ g_{5}(x)=0.125-x_{1}\leq 0\\ g_{6}(x)=Q-Q_{c}(x)\leq 0\\ g_{7}(x)=1.10471x_{1}^{2}+0.04811x_{3}x_{4}\left(14+x_{2}\right)-5\leq 0 \end{array}$$

where the parameters and intermediate expressions are

$$\begin{array}{r} Q=6000\;lb,\;L=14in,\;D=30\times 10^{6\;}psi,\;S=12\times 10^{6\;}psi\\ H_{max}=136,000\;psi,\;\delta_{max}=0.25in\\ 0.1\leq x_{1},x_{4}\leq 2,\;0.1\leq x_{2},x_{3}\leq 10 \end{array}$$

Supporting equations include

$$\begin{array}{r} H(x)=\sqrt{H^{\prime 2}+2H^{\prime }H^{\prime \prime }\frac{x_{2}}{2R}+H^{\prime \prime 2}}\\ H^{\prime }=\frac{Q}{\sqrt{2}x_{1}x_{2}},\;H^{\prime \prime }=\frac{MR}{J}\\ M=Q\left(L+\frac{x_{2}}{2}\right),\;R=\sqrt{\frac{x_{2}^{2}}{4}+{\left(\frac{x_{1}+x_{3}}{2}\right)}^{2}}\\ J=2\left(\frac{x_{2}^{2}}{12}+{\left(\frac{x_{1}+x_{3}}{2}\right)}^{2}\right)\sqrt{2}x_{1}x_{2}\\ M(x)=\frac{6QL}{x_{4}x_{3}^{2}},\;\delta (x)=\frac{4QL}{Dx_{4}x_{3}^{3}},\\ Q_{c}(x)=\left(1-\frac{x_{4}^{3}}{2L}\sqrt{\frac{D}{4S}}\right)\left(\frac{4.013\sqrt{DSx_{3}^{2}x_{4}^{6}/36}}{L^{2}}\right) \end{array}$$

The mathematical model of three-bar truss can be described as follows:

$$minf(x)=\left(2\sqrt{2}x_{1}+x_{2}\right)\cdot l$$

Subject to the stress constraints:

$$\begin{array}{rr} & g_{1}(x)=\frac{\sqrt{2x_{1}+x_{2}}}{\sqrt{2}x_{1}^{2}+2x_{1}x_{2}}Q-H\leq 0\\ & g_{2}(x)=\frac{x_{2}}{\sqrt{2}x_{1}^{2}+2x_{1}x_{2}}Q-H\leq 0\\ & g_{3}(x)=\frac{1}{\sqrt{2}x_{2}+x_{1}}Q-H\leq 0 \end{array}$$

Design variable bounds are

$$0\leq x_{1},x_{2}\leq 1$$

Problem constants include

$$l=100\;cm,Q=2kN/{cm}^{2},H=2kN/{cm}^{2}$$

The fitness function for UAV path planning can be described as follows:

The path planning problem of UAVs aims at calculating a trajectory that fulfills the minimum cost of the whole mission which depends on two main factors, the risk accumulated along the flight path and the effort necessary to accomplish the mission. Both factors are expressed as integrals with respect to the path length, measuring the way in which the threat exposure and fuel consumption change throughout the route that the UAV takes. The threat cost $J_{\mathrm{threat}\;}$ and the fuel cost $J_{\mathrm{fuel}\;}$ are formulated as

(A1) $$\begin{array}{r} J_{\mathrm{threat}\;}=\int_{0}^{\mathrm{length}}\;w_{\mathrm{threat}\;}dl\\ J_{\mathrm{fuel}\;}=\int_{0}^{\mathrm{length}\;}\;\;w_{\mathrm{fuel}\;}dl \end{array}$$

where $w_{\mathrm{threat}\;}$ and $w_{\mathrm{fuel}\;}$ represent the cost per unit distance related to risk and energy, respectively, and length denotes the total trajectory length. To improve the fidelity of threat impact evaluation, each path segment connecting two consecutive waypoints is divided into five subsegments of equal length. As depicted in Figure 9, subpoints are placed at 10%, 30%, 50%, 70%, and 90% of the segment length. Each of these subpoints is assessed for its closeness to the center of threat regions. If a subpoint lies within a threat zone, its contribution to the segment’s threat cost is determined using an inverse fourth-power distance function, resulting in a non-linear penalty. The threat cost for the i-th segment is calculated as

(A2) $$w_{\mathrm{threat},L_{i}}=\frac{{\;\mathrm{length}\;}_{i}}{5}\sum_{k=1}^{N_{\mathrm{threat}\;}}\;t_{k}\left(\frac{1}{d_{0.1,i,k}^{4}}+\frac{1}{d_{0.3,i,k}^{4}}+\frac{1}{d_{0.5,i,k}^{4}}+\frac{1}{d_{0.7,i,k}^{4}}+\frac{1}{d_{0.9,i,k}^{4}}\right)$$

Here, $N_{\mathrm{threat}\;}$ represents the number of threat regions, $t_{k}$ is the intensity level of the k-th threat, and $d_{x,i,k}$ is the Euclidean distance from the subpoint at fraction x of segment i to the center of the k-th threat zone. The fuel cost component is computed by summing the distances between each pair of consecutive nodes along the path and includes the final leg from the last waypoint to the destination:

(A3) $$w_{\mathrm{fuel}\;}=\sum_{k=1}^{N}\;\left(d_{k,k+1}+d_{k+1,g}\right)$$

In this formulation, $d_{k,k+1}$ denotes the distance between nodes k and k+1, and $d_{k+1,g}$ indicates the remaining distance to the goal from the k+1-th node. To combine these two cost components, a weighted objective function is employed, balancing the need to minimize energy use with the imperative to avoid high-risk areas. The total mission cost is defined as

(A4) $$J_{\cos t\;}=\alpha \cdot J_{\mathrm{threat}\;}+(1-\alpha )\cdot J_{\mathrm{fuel}\;}$$

The weighting factor α∈(0,1) governs the emphasis of the optimization process: values of α close to 0 prioritize shorter, more energy-efficient paths, while values closer to 1 focus more on minimizing threat exposure. In the experiments conducted in this study, α is set to 0.5, equally valuing safety and efficiency to represent a balanced mission strategy.
